# DOT1L regulates chamber-specific transcriptional networks during cardiogenesis and mediates postnatal cell cycle withdrawal

**DOI:** 10.1038/s41467-022-35070-2

**Published:** 2022-12-02

**Authors:** Paola Cattaneo, Michael G. B. Hayes, Nina Baumgarten, Dennis Hecker, Sofia Peruzzo, Galip S. Aslan, Paolo Kunderfranco, Veronica Larcher, Lunfeng Zhang, Riccardo Contu, Gregory Fonseca, Simone Spinozzi, Ju Chen, Gianluigi Condorelli, Stefanie Dimmeler, Marcel H. Schulz, Sven Heinz, Nuno Guimarães-Camboa, Sylvia M. Evans

**Affiliations:** 1 Skaggs School of Pharmacy and Pharmaceutical Sciences, University of California San Diego, 92093 La Jolla, CA USA; 2 Institute of Genetic and Biomedical Research (IRGB), Milan Unit, National Research Council of Italy, 20138 Milan, Italy; 3 Institute of Cardiovascular Regeneration, Center of Molecular Medicine, Goethe University, 60590 Frankfurt am Main, Germany; 4 German Center for Cardiovascular Research (DZHK), Partner Site Rhein Main, 60590 Frankfurt am Main, Germany; 5 Cardiopulmonary Institute, Goethe University Frankfurt, 60590 Frankfurt am Main, Germany; 6 Department of Medicine, University of California San Diego, 92093 La Jolla, CA USA; 7 Faculty of Biological Sciences, Goethe University, 60590 Frankfurt am Main, Germany; 8 IRCCS Humanitas Research Hospital, 20089 Rozzano (MI), Italy; 9 Department of Medicine, Meakins-Christie Laboratories, McGill University, H4A 3J1 Montreal, QC Canada; 10 Department of Biomedical Sciences, Humanitas University, 20090 Pieve Emanuele (MI), Italy

**Keywords:** Organogenesis, Cardiovascular biology, Histone post-translational modifications, Chromatin remodelling, Cell-cycle exit

## Abstract

Mechanisms by which specific histone modifications regulate distinct gene networks remain little understood. We investigated how H3K79me2, a modification catalyzed by DOT1L and previously considered a general transcriptional activation mark, regulates gene expression during cardiogenesis. Embryonic cardiomyocyte ablation of *Dot1l* revealed that H3K79me2 does not act as a general transcriptional activator, but rather regulates highly specific transcriptional networks at two critical cardiogenic junctures: embryonic cardiogenesis, where it was particularly important for left ventricle-specific genes, and postnatal cardiomyocyte cell cycle withdrawal, with Dot1L mutants having more mononuclear cardiomyocytes and prolonged cardiomyocyte cell cycle activity. Mechanistic analyses revealed that H3K79me2 in two distinct domains, gene bodies and regulatory elements, synergized to promote expression of genes activated by DOT1L. Surprisingly, H3K79me2 in specific regulatory elements also contributed to silencing genes usually not expressed in cardiomyocytes. These results reveal mechanisms by which DOT1L successively regulates left ventricle specification and cardiomyocyte cell cycle withdrawal.

## Abstract (web summary)

How and whether histone modifications regulate distinct gene networks remains insufficiently understood. Here Cattaneo et al show that DOT1L catalyzed H3K79me2 regulates fetal chamber-specific gene expression and neonatal cardiomyocyte cell cycle withdrawal to coordinate heart development.

## Introduction

Epigenetic enzymes play critical roles in organogenesis by defining chromatin structures required for cell type-specific transcriptional networks^1^. Yet, mechanisms by which genome-wide histone modifications result in activation or repression of specific genes remain little understood. DOT1L is the only epigenetic enzyme catalyzing methylation of lysine 79 of histone 3 (H3K79me)^2,3^. Originally identified in yeast due to its role in maintenance of telomeric regions^2^, DOT1L has been extensively studied in MLL-rearranged leukemia, where it is considered an emerging therapeutic target^4^. Initial genome-wide studies suggested that, rather than being associated with specific gene expression programs, H3K79me2/3 is ubiquitously found in all transcribed loci, leading to a model of H3K79me as a generic mark of active genes^5^.

Recent studies have revealed that, in addition to gene body H3K79me, DOT1L can regulate expression of its targets via methylation of regulatory regions^6^. We have previously shown that, in vitro, DOT1L is required for proper differentiation of embryonic stem cells into cardiomyocytes (CMs)^7^, however it is currently unclear whether this enzyme is necessary for normal cardiogenesis in vivo. In mice, global ablation of *Dot1l* results in embryonic lethality between E9.5 and E10.5 with mutants displaying yolk sac angiogenic defects, growth impairment and cardiac dilation^8^. Selective *Dot1l* ablation in CMs using *αMHC-Cre* results in an adult lethal phenotype with perturbations in dystrophin expression^9^. However, owing to the nature of this knockout (using a Cre that is not active during early embryogenesis^10,11^), it is not known whether DOT1L plays any role in cardiogenesis. Furthermore, our molecular understanding of the functioning of this enzyme in cardiogenesis in vivo is limited by the absence of RNA-seq or ChIP-seq datasets that would allow for unbiased identification of direct targets of DOT1L in embryonic or neonatal CMs.

The mammalian heart develops from two major populations of cardiogenic progenitors – the first and second heart fields. Cells from the first heart field give rise to the left ventricle, whereas cells from the second heart field give rise to the right ventricle and most of the atria^12^. A set of asymmetrically expressed transcription factors are essential for the establishment of chamber-specific transcriptional programs that regulate cardiac patterning and chamber maturation^13–16^. Amongst these, *Irx4* (expressed only in ventricular CMs but absent from the atria) is essential to establish a ventricular identity in lieu of an atrial phenotype^17^. *Hand1* (expressed in the left ventricle throughout development and in the cardiac conduction system post-birth^18^) and *Hand2* (restricted to the right ventricle in early patterning and subsequently expressed in endocardium and myocardium of both ventricles) play a major role in defining systemic and pulmonary ventricular identity, respectively^19–21^. Epigenetic mechanisms contributing to the tightly regulated expression of chamber-specific genes remain mostly unexplored, however, it is known that the H3K4 methyltransferase SMYD1/BOP1 is a critical regulator of *Hand2* expression^22^. To date, it is not known whether any particular epigenetic enzyme plays a similar role in left ventricle-specific regulation of *Hand1* expression.

Regulated CM proliferation is a major component of normal cardiogenesis. The rate of CM mitosis gradually decreases from midgestational stages until birth, reaching a state of complete mitotic withdrawal in the first week after birth, when the majority of CMs become binucleated^23^. Therefore, a major goal in cardiac regeneration is the identification of strategies to promote proliferation of adults CMs.

To test whether DOT1L plays a role in cardiogenesis, we ablated a floxed *Dot1l* allele using a Cre allele that is active in CMs from early developmental timepoints and observed a perinatal lethal phenotype with abnormal cardiac morphology. This phenotype resulted from perturbations in highly specific gene expression programs that orchestrate normal cardiogenesis at two distinct timepoints: embryonic heart development and neonatal CM cell cycle withdrawal. In embryogenesis, DOT1L emerged as a major regulator of the expression of several transcription factors involved in chamber-specific gene expression profiles, with left ventricle-specific genes being particularly sensitive to the absence of this enzyme. In the neonatal period, DOT1L promoted expression of genes involved in CM maturation and cell cycle exit, with Dot1L knockouts exhibiting sustained CM proliferation, a feature of interest for cardiac regeneration. Integration of CM ChIP-seq and Hi-C data revealed that DOT1L regulated target genes via two mechanisms: methylation of H3K79 in gene bodies and methylation of H3K79 in regulatory elements (K79-REs). Our analyses identified two types of K79-REs: activating elements that synergized with gene body H3K79me2 to promote expression of targets activated by DOT1L, and K79-REs that contributed to silencing of genes normally not expressed in CMs. In contrast to H3K79me2 being a general activator of gene transcription as previously thought, our results reveal H3K79me2 as an epigenetic mark that regulates highly specific gene programs by both activating and repressing target genes.

## Results

### Abnormal cardiogenesis in DOT1L cKOs

**Fig. 1:**
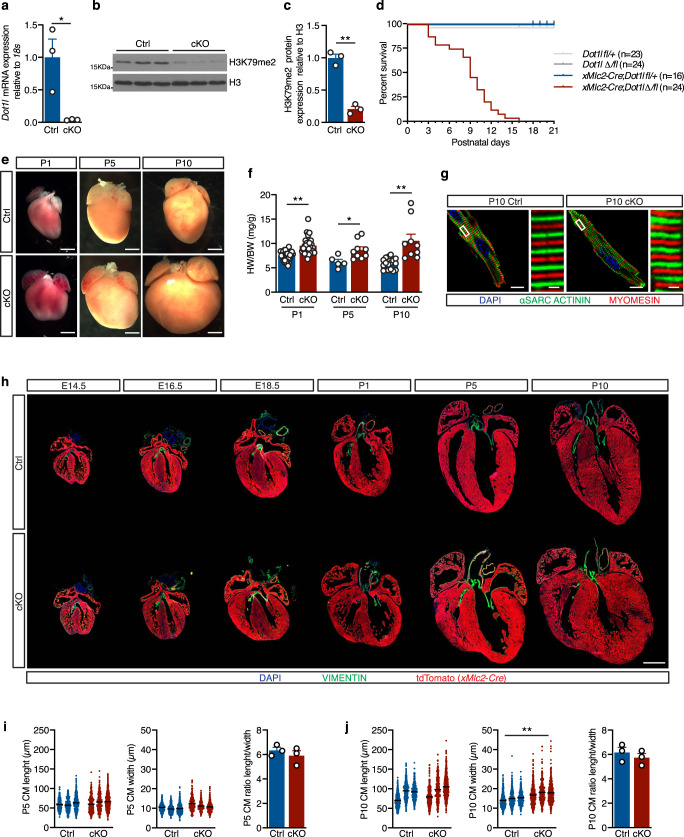
Cardiomyocyte-specific ablation of DOT1L from early developmental timepoints results in enlarged hearts and peri-natal lethality. **a** qPCR analysis using a primer within the floxed exon of *Dot1l* mRNA showing efficient ablation of this gene in E12.5 cKO FACS-sorted CMs (*N* = 3 biological replicates, unpaired *t*-test, two-sided *P* = 0.0271). Western blot (**b**) and respective quantification (**c**) showing strongly reduced H3K79me2 levels in E14.5 hearts upon ablation of Dot1L (*N* = 3 biological replicates, unpaired *t*-test, two-sided *P* = 0.0004). **d** Kaplan–Meier survival curves showing postnatal lethality of Dot1L cKOs. **e** Whole mount images of postnatal day (P) 1, P5 and P10 hearts in Ctrls and Dot1L cKOs representative of the enlarged heart phenotype of Dot1L cKOs (scale bars = 1 mm). **f** Graph representing a significant increase in heart weight/body weight ratio (HW/BW (mg/g)) in Dot1L cKO vs Ctrl in all stages analyzed. (P1 Ctrl *N* = 36, P1 cKO *N* = 30, unpaired *t*-test, two-sided *P* < 0.0001; P5 Ctrl *N* = 5, P5 cKO *N* = 9, unpaired *t*-test, two-sided *P* = 0.0223; P10 Ctrl *N* = 16, P10 cKO *N* = 9 biological replicates, unpaired *t*-test, two-sided *P* = 0.0002). **g** Confocal images showing no alterations in sarcomere organization and myofiber orientation in Dot1L cKOs. αSarcomeric Actinin in green, Myomesin in red, DAPI (4’,6-diamidin-2-phenylindol) in blue (scale bar = 10 μm for lower magnification images on the left and 1 μm for higher magnification images on the right). **h** Immunofluorescence time course depicting the dynamics of phenotypic manifestations in Dot1L cKOs in embryonic (E) and postnatal (P) stages. DAPI in blue, Vimentin in green and lineage traced *xMlc2*-Cre;tdTomato CMs in red (scale bar = 1 mm). Assessment of CM length (left), width (middle) and ratio of CM length/width (right) at P5 (**i**) and P10 (**j**) indicated no major changes in CM size. Measurements were performed on isolated CMs (mean of 413 CMs counted per heart from *N* = 3 biological replicates, unpaired *t*-test, two-sided *P* = 0.0096 for P10 CM width). In all graphs Ctrl indicates control mice (*XMlc2-Cre;Dot1L fl/+)*, cKO indicates mutant mice (*XMlc2-Cre;Dot1L Δ/fl*). Data is presented as mean ± SEM; * represents *P* ≤ 0.05, **P ≤ 0.01. Source data are provided as a Source Data file.

Following our previous observation that DOT1L is required for the differentiation of embryonic stem cells into CMs^7^, we decided to assess if this enzyme is required for normal cardiogenesis in vivo. Fluorescent RNA in situ hybridization studies showed that *Dot1l* is robustly expressed in CMs at least from E10.5 onwards (Supplementary Fig. 1a). This expression was observed throughout the heart, without being specific to any chamber. *Dot1l* expression was also observed in non-cardiomyocyte cells of the heart, as well as most tissues outside the heart, showing this enzyme is not cardiac-specific. However, at E16.5, *Dot1l* expression levels were higher in CMs than in the developing valves (Supplementary Fig. 1b). *Dot1l* has been previously ablated in the heart using an *αMHC-Cre* line, leading to an adult lethal phenotype with reduced dystrophin expression^9^. We have shown that *αMHC-Cre* is not optimal for myocardial-restricted knockouts due to its expression in non-myocyte lineages inside and outside the heart, and because it does not flox-out in all CMs at an early time point^10,11^. To avoid these limitations and study CM-specific roles of DOT1L from early embryonic timepoints, we ablated a floxed *Dot1l* allele (loxP sites flanking exon 2) using the *xMlc2-Cre* allele^24^ that drives highly specific and efficient recombination in CMs (Supplementary Fig. 1c) from the cardiac crescent stage^24^. In our analyses, we compared *xMlc2-Cre*+; *Dot1l-WT/flox* mice (herein designated as Dot1L Ctrl or controls) with *xMlc2-Cre*+; *Dot1l-Δ/flox* littermate mice (herein designated as Dot1L cKO or mutants). Inclusion of a copy of the *Rosa26-tdTomato* reporter allele^25^ in all crosses allowed highly specific labeling of CMs by the red fluorescent protein tdTomato for downstream flow cytometry and confocal microscopy applications (Supplementary Fig. 1d). Real time qPCR analyses revealed highly efficient ablation of the floxed allele in FACS-sorted E12.5 CMs (Fig. 1a).

DOT1L is the only enzyme catalyzing H3K79 methylation^3^. Consequently, CM ablation of *Dot1l* resulted in a strong reduction in H3K79me2 levels in E14.5 hearts (Fig. 1b, c). CM-specific ablation of *Dot1l* did not result in an embryonic lethal phenotype, as animals with the mutant genotype were observed at expected Mendelian ratios in all stages analyzed (Supplementary Fig. 1e). Despite being born at expected numbers, Dot1L cKO mice started dying shortly after birth, with 50% mortality by postnatal day 9 (P9), and the longest-lived mutant surviving until P16 (Fig. 1d). Macroscopic examination of organs at neonatal stages revealed that mutants had enlarged hearts with a rounded shape relative to controls (Fig. 1e). This phenotype was exacerbated as animals aged and peaked at P10 in surviving animals (Fig. 1e). This evident cardiac enlargement translated into increased heart weight (Supplementary Fig. 1f) and increased heart weight/body weight ratios (Fig. 1f) without changes in body weight (Supplementary Fig. 1g). Co-immunostaining for αSarcomeric Actinin and Myomesin revealed absence of myofiber orientation defects in CMs from Dot1L cKO hearts (Fig. 1g).

To determine the timing of onset of the cardiac phenotype and assess for additional morphogenic malformations, we conducted a histological time course analysis spanning from midgestation to postnatal timepoints (Fig. 1h). cKO hearts were completely indistinguishable from control littermates until E14.5 and started exhibiting the enlarged, rounded phenotype between E16.5 and E18.5. Postnatally, mutant hearts displayed ventricular walls with increased thickness and areas of moderate persistent trabeculation (Fig. 1h). No additional major morphogenic abnormalities (septal or outflow defects) were observed. Immunostaining for Vimentin revealed absence of valve malformations and absence of major foci of fibrosis in cKOs (Fig. 1h). Absence of fibrotic remodeling was also confirmed by qPCR for fibrotic markers *Col1a1* and *Col3a1* (Supplementary Fig. 2a) performed on whole heart tissue, immunostaining for Collagen1 (Supplementary Fig. 2b), and Masson trichrome staining (Supplementary Fig. 2c). To assess if the cardiac enlargement observed in cKOs is a consequence of CM hypertrophy, we conducted length and width measurements of CMs isolated from Dot1L cKO and control hearts. These analyses revealed that, at P5, CMs from both genotypes had comparable sizes (Fig. 1i). At P10, CM length was similar in both groups, but CM width was moderately increased in cKOs, without causing a significant change in the CM length-to-width ratio (Fig. 1j). These results ruled out CM hypertrophy as a cause of the observed cardiac enlargement.

**Fig. 2:**
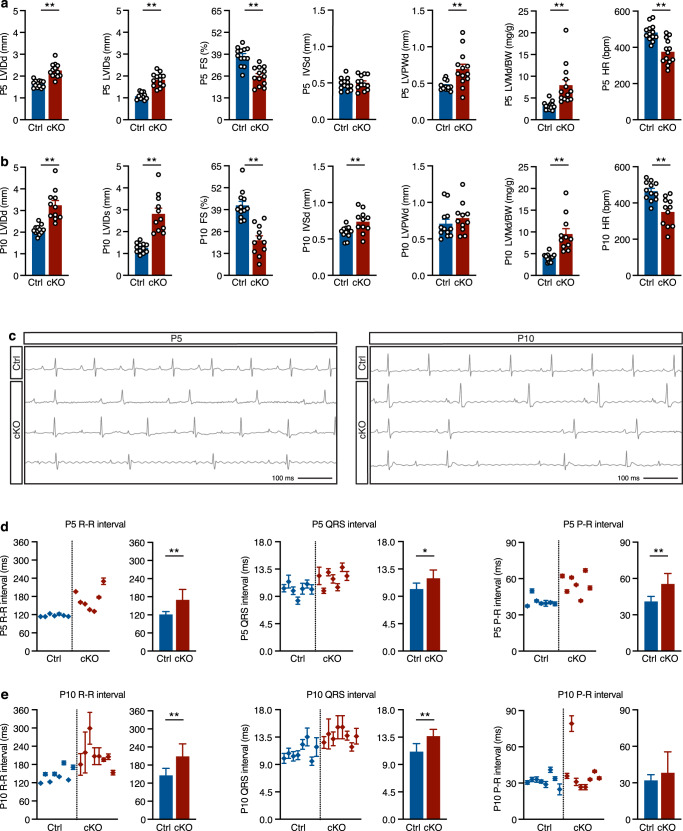
Echocardiographic and electrocardiographic defects in Dot1L cKO hearts. Echocardiographic analyses conducted at P5 (**a**) and P10 (**b**) revealed significant defects in Dot1L cKO hearts at both timepoints, including increased left ventricular inner diameter, both in diastole (LVIDd) and systole (LVIDs), reduced fractional shortening (FS), and increased diastolic left ventricle mass to body weight ratios (LVMd/BW). Dot1L cKOs also exhibited increased diastolic left ventricular posterior wall thickness (LVPWd) at P5 and increased diastolic intra-ventricular septum thickness (IVSd) at P10. Additionally, Dot1L cKOs displayed reduced heart rate (HR) at both timepoints. (P5 Ctrl *N* = 13, P5 cKO *N* = 13, P10 Ctrl *N* = 12, P10 cKO *N* = 11 biological replicates; unpaired *t*-test, two-sided P5 LVIDd *P* < 0.0001, P5 LVIDs *P* < 0.0001, P5 FS *P* < 0.0001, P5 LVPWd *P* = 0.0029, P5 LVMd/BW *P* = 0.0017, P5 HR *P* < 0.0001; P10 LVIDd *P* < 0.0001, P10 LVIDs *P* < 0.0001, P10 FS *P* < 0.0001, P10 IVSd *P* = 0.0083, P10 LVMd/BW *P* = 0.0002, P10 HR *P* < 0.0005). **c** Representative ECG tracks from Ctrl and Dot1L cKO. ECG measurements revealed multiple defects in Dot1L cKOs both at P5 (**d**) and P10 (**e**), including increased and irregular R-R and QRS intervals. At P5 Dot1L cKOs also displayed significantly increased P-R intervals, however, this difference could not be detected in P10 mutants. (P5 *N* = 7, P10 *N* = 8 biological replicates, unpaired *t*-test, two-sided P5 R-R *P* = 0.004, P5 QRS *P* = 0.0164, P5 P-R *P* = 0.0017, P10 R-R *P* = 0.0024, P10 QRS *P* = 0.001, P10 P-R *P* = 0.2279). The left part of the graph represents the mean ± SD of multiple beats measured for each mouse. The right graph represents the mean ± SD of multiple biological replicates. Data is presented as mean ± SD; * represents *P* ≤ 0.05, **P ≤ 0.01. Source data are provided as a Source Data file.

Echocardiographic assessment of heart function revealed that, compared with littermate controls, Dot1L cKO mice had reduced fractional shortening both at P5 and P10, increased left ventricular posterior wall thickness and increased left ventricular chamber diameter (Fig. 2a, b). Electrocardiographic analyses revealed that Dot1L cKO mutants had reduced heart rate both at P5 and P10, which translated into prolonged and irregular R-R intervals, with occasional beat drops (Fig. 2c–e). At P5, Dot1L cKOs also had prolonged QRS and P-R intervals, whereas at P10 the P-R intervals of mutants were comparable to those of controls, suggesting that the mutants with more severe conduction defects might die between P5 and P10. Despite the strong cardiac phenotype, Dot1L cKOs presented no obvious signal of distress prior to death. Therefore, it is possible that defects in cardiac conduction might account for the sudden lethal phenotype.

Altogether, these findings identify a critical role for DOT1L in normal cardiogenesis and suggest that the strong macroscopic and functional phenotypes observed in Dot1L cKOs are not a consequence of fibrotic remodeling or major alterations in CM structure or size.

### DOT1L is essential for chamber-specific gene expression

**Fig. 3:**
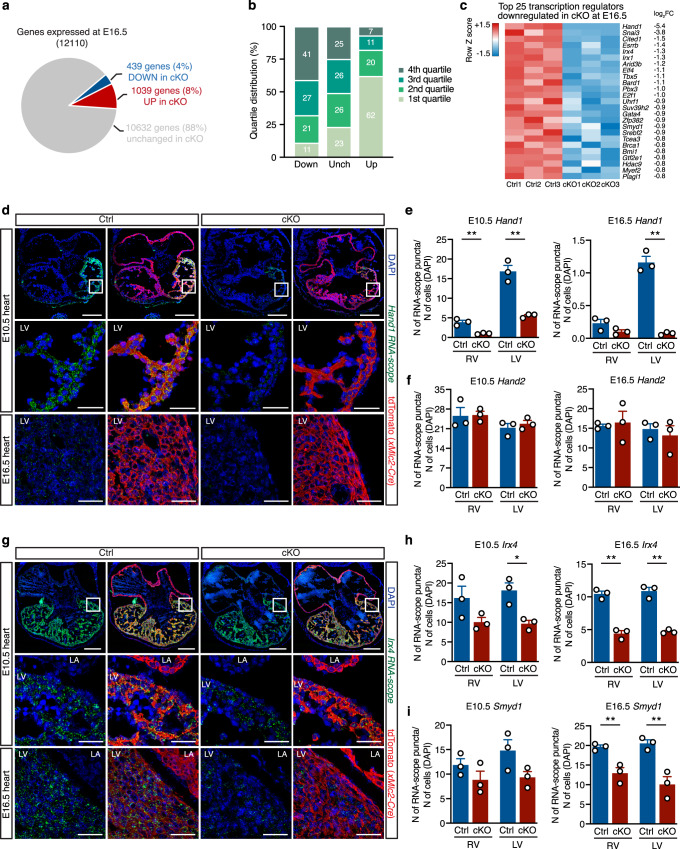
DOT1L is required in cardiomyocytes for chamber-specific gene expression. **a** Pie chart representing the number of genes down- (log_2_FC ≤ −0.5; FDR ≤ 0.05) and up-regulated (log_2_FC ≥ 0.5; FDR ≤ 0.05) in Dot1L cKO CMs at E16.5. **b** Quartile distribution of gene expression in CMs at E16.5. Genes downregulated (Down) in Dot1L cKO were expressed at a high level in control CMs, whereas the majority of upregulated genes (Up) belonged to the bottom quartile of expression. Genes not significantly modulated (Unch) were evenly distributed across quartiles of expression. Data are shown as stacked percentage bar graph. **c** Heatmap showing the expression of the top 25 transcription regulators downregulated in Dot1L cKO CMs at E16.5, highlighting that multiple chamber-specific transcription regulators were significantly downregulated. RNA-scope analyses (**d**) and respective quantification (**e**) validating blunted expression of *Hand1* in Dot1L cKOs both at E10.5 and E16.5. (*N* = 3 biological replicates; unpaired *t*-test, two-sided, E10.5 RV *P* = 0.0042, E10.5 LV *P* = 0.0020, E16.5 LV *P* = 0.0003). **f** Quantification of RNA-scope analysis validating no changes in expression of *Hand2* in Dot1L cKOs both at E10.5 and E16.5 (RNA-scope images presented in Supplementary Fig. 4) (*N* = 3 biological replicates). RNA-scope analyses (**g**) and respective quantification (**h**) validating reduced levels of *Irx4* in Dot1L cKOs both at E10.5 and E16.5. (*N* = 3 biological replicates; unpaired *t*-test, two-sided, E10.5 LV *P* = 0.0153, E16.5 RV *P* = 0.0006, E16.5 LV *P* = 0.0005). **i** Quantification of RNA-scope analysis validating reduced levels of *Smyd1* in Dot1L cKOs both at E10.5 and E16.5 (RNA-scope images presented in Supplementary Fig. 3). (*N* = 3 biological replicates; unpaired *t*-test, two-sided, E16.5 RV *P* = 0.0057, E16.5 LV *P* = 0.008). For panels **d** and **g** scale bars = 250 μm for low magnification panels and 50 μm for high magnification images. In panels **e**, **f**, **h** and **i** data is presented as mean ± SEM; * represents *P* ≤ 0.05, **P ≤ 0.01. (*N* = 3 biological replicates). Source data are provided as a Source Data file.

To decipher gene expression networks misregulated in the absence of DOT1L, we used RNA-seq to assess the transcriptomes of FACS-sorted Ctrl and cKO CMs just prior to the onset of clear phenotypic changes (E16.5). An average of 100,000 CMs were sorted per E16.5 heart (including CMs from all four cardiac chambers), yielding enough RNA to prepare biological replicates from individual hearts (3 replicates per genotype group). Bioinformatics analyses revealed that, from a total of 12,110 expressed genes (RPKM ≥ 1 in either Ctrl or cKO), 1478 were misregulated in cKO versus Ctrl CMs (log_2_FC ≤ −0.5 or ≥0.5, false discovery rate ≤0.05). From these, 439 genes were downregulated and 1039 upregulated in cKOs (Fig. 3a and Supplementary Data 1a). Transcripts upregulated in cKOs corresponded to genes that are normally expressed at low levels in control CMs (62% of genes in the lower expression quartile), whereas downregulated genes corresponded to genes normally expressed at a medium/high level (Fig. 3b). Notably, the gene displaying the highest downregulation (−5.4 log_2_FC) in mutant CMs was the transcription factor *Hand1*, a central element in the establishment and maintenance of left ventricular identity (Fig. 3c and Supplementary Data 1a). In addition, amongst genes significantly downregulated in mutants there were several other genes asymmetrically expressed across the cardiac chambers: the transcription factors *Irx4* (ventricular specific^17^), *Tbx5* (left ventricle and atrial-specific^26^), and the left ventricular-specific genes *Gja5* (encoding Connexin40) and *Cited1* (Fig. 3c and Supplementary Data 1a). These results revealed that transcriptional networks operating in left ventricular CMs (those derived from the first heart field) are particularly sensitive to the absence of DOT1L. In addition to chamber-specific genes, DOT1L also regulated critical cardiogenic factors that are expressed throughout the heart. Examples of these are the transcription factors *Gata4* and *Mef2c*, as well as the epigenetic enzyme *Smyd1*. The transcription factor *Nkx2-5* also exhibited a trend toward downregulation (Supplementary Data 1a). These transcriptomic differences revealed that DOT1L activity in embryonic CMs is essential for establishment of gene expression networks that coordinate normal cardiogenesis. This requirement, however, was not generalized to all cardiac patterning genes, as transcript levels for *Hand2*, *Tbx20* and *Nppa* (ANF) were not altered in mutants (Supplementary Data 1a).

To validate RNA-seq results, we performed fluorescent RNA in situ hybridization (RNA-scope) in histological sections of control and cKO hearts. Strikingly, at E16.5, *Hand1* transcripts were completely undetectable in the left ventricle of mutants (Fig. 3d, bottom panel, quantification in Fig. 3e, right). Downregulation of *Hand1* was already evident at E10.5 when levels of *Hand1* expression in control hearts were higher than at E16.5 (Fig. 3d, top and middle panels, quantification in Fig. 3e, left), indicating that transcriptional consequences of DOT1L ablation preceded the first phenotypic manifestations by several days. Transcript levels for the ventricular-specific transcription factor *Irx4* and for the CM-specific epigenetic modifier *Smyd1* were significantly reduced in cKO hearts both at E10.5 and E16.5 (Fig. 3g and Supplementary Fig. 3, quantification in Fig. 3h, i), confirming RNA-seq results. RNA-scope analyses also showed that Dot1L cKO CMs expressed normal levels of *Hand2*, both at E10.5 and E16.5, further validating that DOT1L regulates specific transcriptional programs, rather than being a generic activator of transcription (Supplementary Fig. 4, quantification in Fig. 3f).

Our data revealed a role for DOT1L in regulating the expression of genes necessary for the establishment and maintenance of chamber identity, in particular those involved in left ventricular identity. While a number of genes were downregulated in cKOs, *Hand1* was particularly sensitive to absence of DOT1L, with *Hand1* transcripts being completely absent from midgestational DOT1L-deficient CMs (both in RNA-seq and RNA-scope). Consistent with these transcriptomic observations, cardiac-specific ablation of *Hand1* phenocopies Dot1L cKO, including an enlarged and round-shaped heart and peri-natal lethality^21^.

### Transcriptional control via gene body H3K79me2

**Fig. 4:**
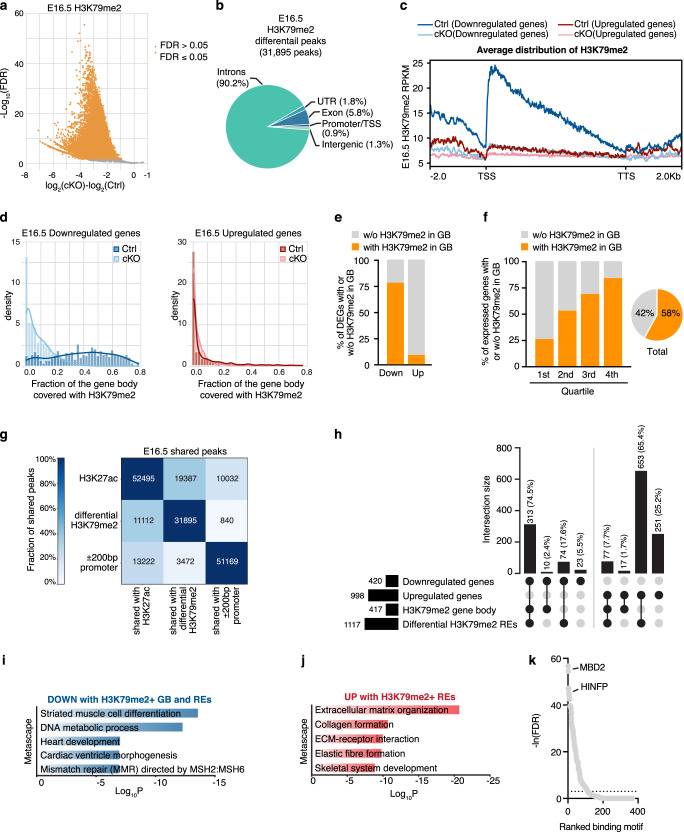
DOT1L controls transcription of target genes via a combination of H3K79me2 in gene bodies and regulatory elements. **a** Volcano plot displaying H3K79me2 ChIP-seq peaks significantly enriched in E16.5 Dot1L cKO vs Ctrl CMs. **b** Pie chart indicating the genomic distribution of differential H3K79me2 ChIP-seq peaks in E16.5 CMs. **c** Metagene profiles showing the average distribution of H3K79me2 input-normalized density relative to Transcription Start Site (TSS) and Transcription Termination Site (TTS) with ±2 Kb flanking regions. **d** Fraction of gene body covered with H3K79me2 in downregulated genes (left graph) and in upregulated genes (right graph). **e** Graph representing the percentage of down- and up-regulated genes in E16.5 cKO CMs with (Coverage ≥ 50 reads and Fraction of gene body ≥ 0.2) or without (Coverage < 50 reads or Fraction of gene body <0.2) gene body H3K79me2 in E16.5 Ctrl CMs. **f** Percentage of genes with H3K79me2 in the gene body (GB) or without H3K79me2 in the gene body across the distinct quartiles of expression. This modification was abundant amongst highly expressed genes (4^th^ quartile of RNA expression) and progressively decreased towards the lower quartiles of expression. Globally more than half (58%) of all genes expressed in E16.5 CMs had gene body H3K79me2. **g** Heatmap indicating the number of total and shared regions between H3K27ac ChIP-seq peaks in Ctrl CMs, differential H3K79me2 ChIP-seq peaks and promoters (±200 bp around 5’TSS) in E16.5 CMs. Different intensities of colors indicate the fraction (%) of shared peaks. **h** UpSet plot indicating the number and percentage of genes up and downregulated in cKOs versus Ctrls with or without H3K79me2 in gene body (GB) and/or regulatory elements (REs) in E16.5 CMs. Metascape pathway analysis of genes downregulated with H3K79me2 in GB and K79-REs (**i**) and upregulated without gene body H3K79me2 but with K79-REs (**j**) in Dot1L cKO versus Ctrl E16.5 CMs. Top 5 enriched categories are shown, sorted by Log_10_ *P* value. **k** Motif enrichment analysis ranking transcription factors (TFs) enriched in K79-REs associated with upregulated genes without H3K79me2 in GB versus K79-REs associated with downregulated genes with H3K79me2 in GB.

Several core cardiac transcription factors are differentially expressed in Dot1L cKO CMs. Thus, genes modulated in our transcriptomic analyses likely include a combination of targets directly regulated by DOT1L as well as those indirectly regulated owing to secondary effects. To identify genes directly regulated by DOT1L, we performed ChIP-seq assays for H3K79me2 in E16.5 control and cKO FACS-sorted CMs. These analyses revealed a total of 31,895 H3K79me2 peaks significantly enriched in control versus Dot1L cKO CMs (Supplementary Data 2a). The fact that the vast majority of H3K79me2 peaks in Ctrls were lost in cKOs is consistent with DOT1L being the sole histone K79 methyltransferase and validated the purity of our sorted CMs (Fig. 4a and Supplementary Data 2a). Genome-wide differential peak distribution analysis revealed that H3K79me2 is mostly an intragenic modification (introns, exons, UTR and promoter/TSS) with only 1% of differential peaks being located in intergenic regions (Fig. 4b).

Genes downregulated in cKOs exhibited, in control CMs, maximal levels of H3K79me2 immediately after the transcription start site (TSS) that progressively decreased towards the transcription termination site (TTS) (Fig. 4c). Gene body H3K79me2 (coverage ≥ 50 reads and fraction of gene body ≥ 0.20 in control CMs) was present in 341 downregulated genes (77% of all downregulated genes) and 98 upregulated genes (9% of all upregulated genes) (Fig. 4d, e and Supplementary Data 1a). This observation suggests that a significant part of gene downregulation observed in cKOs can be directly attributed to H3K79me2 in the gene body, whereas gene upregulation does not seem to be directly associated with gene body H3K79me2. This is consistent with the view of gene body H3K79me2 as an activation mark^5^, however, it should be noted that this modification is not an absolute requirement for transcription, as 42% of all genes expressed in control CMs did not have significant levels of gene body H3K79me2 (Fig. 4f). Within genes showing gene body H3K79me2, there were two clear categories: genes dependent on DOT1L (modulated in cKOs) and genes not significantly affected by the loss of DOT1L, suggesting a scenario in which other epigenetic modifications can compensate for the loss of gene body H3K79me2 in a subset of genes. Notably, the majority of genes whose transcription depended on gene body H3K79me2 belonged to the highest quartiles of expression, suggesting this modification might play a role in supporting high transcriptional levels (Fig. 4f).

### Transcriptional control via H3K79me2 in regulatory elements

Recent studies in leukemia showed that there is a distinct subset of enhancers that depend on H3K79me2^6^. We conducted analyses to investigate whether CMs have H3K79me2-dependent regulatory elements (REs) that, together with gene body H3K79me2, contribute to gene expression differences observed in cKOs. To identify regulatory regions, we generated CM-specific H3K27ac ChIP-seq datasets. At E16.5, control CMs had 52,495 H3K27ac peaks, mainly occupying introns, promoters/TSS or intergenic regions (Supplementary Fig. 5a). From these, 37% overlapped with H3K79me2 peaks (Fig. 4g and Supplementary Fig. 5b). To predict interactions between candidate regulatory elements and target genes, we applied an adapted implementation of the Activity-by-Contact (ABC) model^27,28^. By integrating information on chromatin state (H3K27ac ChIP-seq) and genomic conformation (Hi-C), this method is more accurate at predicting actual interactions between REs and their target genes than methods simply assigning H3K27ac peaks to the closest neighboring gene^27^. Because the promoter of a gene can serve as a RE for a distinct cis gene^29,30^, our analyses also considered interactions between promoters and distal genes. Using the ABC algorithm, our CM-specific H3K27ac ChIP-seq dataset was combined with a publicly available CM Hi-C dataset^31^ (GSM2544836). To ensure we focused on meaningful candidate RE-target gene interactions, we only considered the most relevant interactions identified by the ABC method (adapted ABC-score ≥0.02): 373,610 interactions, corresponding to 47,547 H3K27ac peaks (Supplementary Data 3a and Supplementary Fig. 5c). From these, 36% (16,963 peaks) overlapped with a differential H3K79me2 peak (Supplementary Data 3a and Supplementary Fig. 5c). This large number of CM REs with H3K79me2 signal (from here on designated as K79-REs) could provide an alternative mechanism for gene expression regulation by DOT1L. Of note, about 34% of all K79-REs observed at E16.5 overlapped a promoter (Supplementary Data 3a and Supplementary Fig. 5c).

To assess the relative contribution of H3K79me2 in gene body versus H3K79me2 in REs to the regulation of target genes, we quantified the effect of these variables in the cumulative distribution of log_2_FC values. These analyses revealed that genes with higher fraction of H3K79me2 in the gene body were more downregulated in cKOs than genes with lower (less than 33%) fraction of H3K79me2 in the gene body (Supplementary Fig. 5d and Supplementary Data 4a). Genes with 6 or more K79-REs displayed stronger modulation than those associated with lower numbers of REs (Supplementary Fig. 5e and Supplementary Data 4a). Focusing exclusively on loci with gene body H3K79me2, those that interacted with K79-REs were more downregulated in mutant CMs than those without these elements, indicating that gene body and regulatory element H3K79me2 synergized to potentiate expression of target genes (Supplementary Fig. 5f and Supplementary Data 4a).

The vast majority (74.5%) of the genes downregulated in DOT1L-deficient CMs were associated with both gene body H3K79me2 and at least one K79-RE (Fig. 4h). Interestingly, 17.6% of all downregulated genes were associated with K79-REs in the absence of gene body H3K79me2. Overall, only 5.5% of downregulated genes were not associated with H3K79me2 in gene body or K79-REs, indicating most gene downregulation was a direct consequence of Dot1L cKO. On the other side of the scale, upregulated genes had reduced association with gene body H3K79me2 (as mentioned above), but, surprisingly, 65.4% of upregulated genes were associated with K79-REs in the absence of gene body H3K79me2. This observation suggests a role for DOT1L in gene silencing via H3K79me2-dependent regulatory elements. Functional annotation revealed that genes downregulated in cKOs with H3K79me2 in gene bodies and REs were involved in differentiation of striated muscle and cardiac morphogenesis (Fig. 4i and Supplementary Data 5a). On the other hand, genes upregulated in cKOs and associated with inhibitory K79-REs were related to non-myocyte functions: extracellular matrix organization and skeletal system development (Fig. 4j and Supplementary Data 5b).

To search for cues as to how DOT1L achieves gene silencing via methylation of REs, we screened for enrichment in binding sites for known transcriptional regulators in the K79-REs of genes upregulated without gene body H3K79me2 but with K79-REs versus genes downregulated with gene body H3K79me2 and K79-REs. This analysis revealed that K79-REs associated with genes upregulated in cKOs (silencing K79-REs) were enriched in binding sites for MBD2, a member of the NuRD complex known to play an important role in transcriptional silencing^32^ (Fig. 4k). Enrichment in NuRD complex binding sites in the silencing K79-REs could provide an explanation for how DOT1L promotes silencing of target genes. However, it is also possible that upregulated genes reflect secondary effects (for example downregulation of a transcription regulator with repressor properties) rather than being a direct consequence of DOT1L inactivation.

### H3K27ac relationship to H3K79me2

**Fig. 5:**
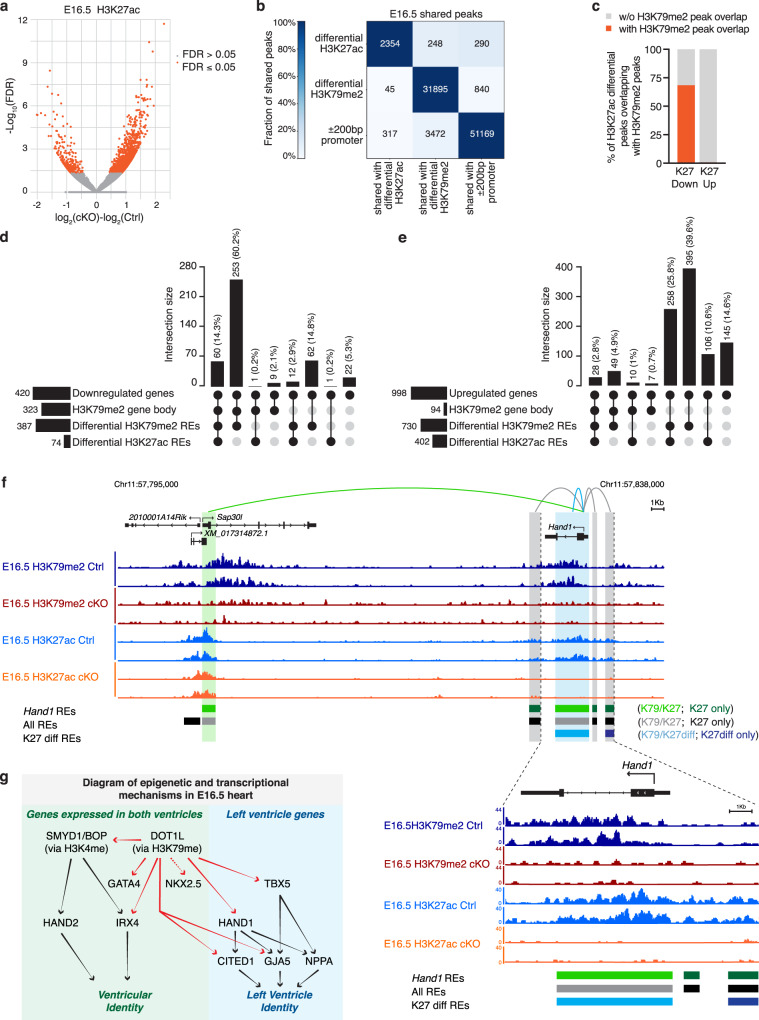
H3K27ac Relationship to H3K79me2. **a** Volcano plot displaying H3K27ac ChIP-seq peaks significantly enriched in E16.5 Dot1L cKO vs Ctrl CMs. **b** Heatmap indicating the number of total and shared regions between differential H3K27ac ChIP-seq peaks, differential H3K79me2 ChIP-seq peaks and promoters (±200 bp around 5’TSS) in E16.5 CMs. Different intensities of colors indicate the fraction (%) of shared peaks. **c** Graph representing the percentage of differential H3K27ac ChIP-seq peaks in E16.5 cKO vs Ctrl CMs overlapping (orange) or not overlapping (gray) with H3K79me2 ChIP-seq peaks. H3K27ac downregulated peaks are more often differential for H3K79me2 than H3K27ac upregulated peaks (oddsratio = 0.004; *p*-value = 5.93e−212). UpSet plots indicating the number and percentage of genes down- (**d**) and upregulated (**e**) in E16.5 Dot1L cKO versus Ctrl CMs with or without H3K79me2 in gene body (GB) and/or regulatory elements (REs) and with or without differential H3K27ac REs. **f** Browser tracks displaying H3K79me2 and H3K27ac ChIP-seq profiles of Ctrl (dark and light blue respectively) and Dot1L cKO (red and orange respectively) E16.5 CMs in the genomic region containing the *Hand1* locus. Loops display all regulatory interactions between REs and the *Hand1* gene, as identified by the ABC analysis. Gray loops identify interactions with REs without H3K79me2, green loops identify interactions with K79-REs, whereas blue loops represent interactions with K79-REs that additionally have H3K27ac REs differentially enriched between Dot1L Ctrls and cKOs. For reference, all REs are displayed in the middle lane in black or gray, regardless of their regulatory association with the *Hand1* gene. Differential H3K27ac REs are indicated in the bottom lane in light blue when they overlap a K79-RE or dark blue when they do not. **g** Diagrammatic representation summarizing the involvement of DOT1L in mammalian cardiogenesis. Genes directly regulated by DOT1L (via H3K79me2) are highlighted in red.

To assess whether H3K27ac in K79-REs is altered upon DOT1L ablation, we also performed H3K27ac ChIP-seq in E16.5 CMs sorted from cKO hearts. Comparison of ChIP-seq profiles from control and mutant CMs identified 2,354 differential H3K27ac peaks (Fig. 5a, b and Supplementary Data 6a). From these, 2,017 were upregulated and 337 downregulated in cKOs (Fig. 5a and Supplementary Data 6a). Most (69%) of the H3K27ac peaks downregulated in cKOs overlapped with a differential H3K79me2 peak, whereas the majority (99%) of upregulated H3K27ac peaks did not (Fig. 5b, c). From the 16,963 K79-REs identified by the ABC method, the vast majority (98%) did not show any difference in H3K27ac enrichment in cKOs, indicating that, at a genome-wide scale, H3K79me2 is not necessary for maintenance of H3K27ac in REs positive for both modifications (Supplementary Data 3a). Despite corresponding to a small fraction (2%) of all K79-REs, those with differential H3K27ac made a relevant contribution to differential gene expression in Dot1L cKOs. From the 313 genes downregulated with H3K79me2 in gene body and K79-REs, 60 (19%) also had differential H3K27ac in at least one RE (Fig. 5d and Supplementary Data 4a). From the 653 upregulated genes without gene body H3K79me2 but with K79-REs, 39% also had differential H3K27ac in at least one RE (Fig. 5e and Supplementary Data 4a). As expected, for K79-REs associated with upregulated genes, differential H3K27ac peaks were mainly upregulated, whereas for K79-REs associated with downregulated genes, differential H3K27ac peaks were mainly downregulated (Supplementary Data 4a). Altogether, these results revealed that, in mutants, differential H3K27ac within a subset of K79-REs may contribute to altered gene regulation by those REs, however, differential H3K27ac is not a prerequisite for gene regulation by K79-REs.

In summary, our mechanistic data revealed that, during embryogenesis, DOT1L directly regulates critical cardiogenic transcription factors: *Hand1*, *Irx4, Gata4*, *Tbx5*, and *Mef2c*, all of which had gene body H3K79me2 combined with K79-REs (Fig. 5g and Supplementary Data 4a). Both *Hand1* and *Irx4* were associated with at least one K79-RE with downregulated H3K27ac in Dot1L cKOs (Fig. 5f, Supplementary Fig. 5g and Supplementary Data 4a). Interestingly, *Smyd1*, encoding the H3K4 methyltransferase necessary for right ventricular expression of *Hand2*^22^, was also a direct target of DOT1L, unveiling complex epigenetic mechanisms for transcriptional regulation in cardiogenesis. Our results also revealed that DOT1L has particular importance in maintenance of left ventricle-specific transcriptional networks, as it directly regulated not only transcription factors specifying the identity of this chamber (*Hand1* and *Tbx5*), but also their direct downstream targets *Cited1* and *Gja5* (Cx40)^21^ (Fig. 5g). On the other hand, *Nppa* (ANF), another classic target of both HAND1^21^ and TBX5^33^ was not altered in Dot1L cKOs, further highlighting the specificity and complexity of this epigenetic control (Fig. 5g). These findings reveal previously unrecognized roles for DOT1L and improve our understanding of the complex transcriptional and epigenetic landscape governing mammalian cardiogenesis.

### Impaired cardiomyocyte cell cycle withdrawal in Dot1L mutants

**Fig. 6:**
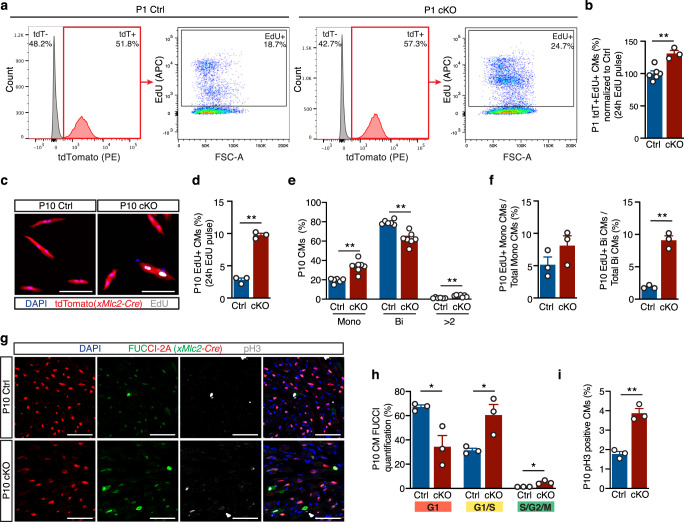
Neonatal Dot1L cKO cardiomyocytes fail to undergo cell cycle withdrawal. Representative FACS analysis (**a**) and respective quantification (**b**) showing significantly increased EdU incorporation within P1 CMs (tdTomato + cells) of Dot1L cKO vs Ctrl hearts (Ctrl *N* = 6, cKO *N* = 3 biological replicates, unpaired *t*-test, two-sided, *P* = 0.0025). Representative immunofluorescence images (**c**), and respective quantification **(d**) showing significantly increased rates of EdU incorporation in P10 CMs isolated from Dot1L cKO vs Ctrl hearts. DAPI in blue, endogenous tdTomato signal driven by *xMlc2*-Cre;Rosa26-tdTomato in red and EdU in white. (Scale bar = 100 μm; Mean of 755 CMs counted per heart from *N* = 3 biological replicates, unpaired *t*-test, two-sided, *P* < 0.0001). **e** Quantification of relative percentage of mononuleated (Mono), binuclated (Bi) or multinucleated (>2) CMs in P10 Dot1L Ctrls and cKOs. At P10, Dot1L cKO hearts had more mononucleated (Mono) and less binucleated (Bi) CMs than littermate Ctrls (mean of 697 CMs counted per heart from *N* = 6 Ctrl and *N* = 7 cKO biological replicates, unpaired *t*-test, two-sided, Mono *P* = 0.0002, Bi *P* < 0.0001, >2 *P* = 0.0007). **f** Quantification of percentage of EdU+ CMs within mononucleated (left graph) and binucleated (right graph) CMs of P10 Dot1L Ctrls and cKOs (Mean of 755 CMs counted per heart from *N* = 3 biological replicates, unpaired *t*-test, two-sided *P* = 0.0006). Immunofluorescence images (**g**) and respective quantification (**h**, **i**) of P10 Dot1L Ctrl and cKO hearts on a *Rosa26-*Fucci2A background. Red-only nuclei represent CMs in G1; red + green (yellow) nuclei represent CMs in G1/S; green-only nuclei correspond to CMs in S/G2/M, phosphor-Histone 3 (pH3) staining in white. Dot1L cKO hearts had a significantly higher percentage of CMs in G1/S and in S/G2/M compared to Ctrls, (**h**, mean of 2070 CMs counted per heart from *N* = 3 biological replicates, unpaired *t*-test, two-sided G1 *P* = 0.0256, G1/S *P* = 0.0308, S/G2/M *P* = 0.0126) and of phopho-Histone3 + (pH3) CMs (**i**, mean of 1019 CMs counted per heart from *N* = 3 biological replicates, unpaired *t*-test, two-sided *P* = 0.0014). (Scale bar = 50 μm; sections have been quantified from all the compartments of the heart). In all graphs from **b** to **i** data is presented as mean ± SEM; * represents *P* ≤ 0.05, **P ≤ 0.01. Source data are provided as a Source Data file.

Defective expression of core cardiogenic factors (including chamber-specific genes) can account for the abnormal morphology of Dot1L cKO hearts, however, it does not explain the increased wall thickness observed in postnatal mutant hearts (Fig. 1h). Dot1L cKO and Ctrl CMs had comparable sizes (Fig. 1i, j), therefore increased wall thickness in mutants was not caused by CM hypertrophy. As increased myocardial wall thickening coincided with the developmental period in which CMs withdraw from cell cycle^23^, we hypothesized this process might be affected in Dot1L cKOs. Flow cytometry analyses of cells isolated from P1 hearts revealed that, at this stage, about 50% of all cardiac cells were CMs (labeled by expression of the reporter gene tdTomato, Fig. 6a). Assessment of EdU incorporation rates (24 h EdU pulse) revealed that, in control hearts, 19% of all CMs were proliferative, whereas this number increased to 26% in mutant hearts (Fig. 6a), representing a statistically significant increase in CM proliferation (Fig. 6b). In mice, by P10 most CMs are withdrawn from cell cycle and 80% of CMs are binucleated^23^. Consistently, fluorescent microscopy analyses of CMs isolated from P10 hearts revealed that less than 3% of control CMs were EdU+(24 h EdU pulse, Fig. 6c, d), compared to 10% in mutant hearts (Fig. 6c, d). Nucleation analysis revealed that Dot1L cKO hearts had almost twice as many mononucleated CMs (34% in cKOs versus 19% in controls) at the expense of binucleated CMs (62% in cKOs versus 80% in Ctrls) (Fig. 6e). Quantification of proliferation ratios across different nucleation categories (Fig. 6f) revealed that Dot1L cKOs had a moderate increase in the percentage of EdU+ mononucleated CMs (8.1% in cKOs versus 5.2% in Ctrls) and a significant 4.9-fold increase in the percentage of EdU+ binucleated CMs (9.1% in cKOs versus 1.86% in Ctrls).

To validate these analyses, we assessed proliferation of control and Dot1L cKO CMs using the *Rosa26-Fucci2a* cell cycle reporter^34^. Expression from this allele is Cre-dependent (thus, restricted to CMs in our analyses) and labels G1 CMs in red, and actively proliferating cells in yellow (G1/S) or green (S/G2/M)^34^. Analysis of histological sections of P10 hearts (Fig. 6g, h and Supplementary Fig. 6a, d) revealed that in controls the majority of CMs (67%) were in G1. G1/S CMs could also be detected (32%), but S/G2/M cells were rare (1%). On the other hand, Dot1L mutant CMs showed significantly different cell cycle distribution across all cycling phases: 34% in G1, 61% in G1/S and 5% in S/G2/M (Fig. 6g, h and Supplementary Fig. 6a, d). In addition, increased proliferation of mutant CMs was also confirmed by phospho-Histone 3 (pH3) antibody staining in histological sections (Fig. 6g, i and Supplementary Fig. 6b, c), as well as EdU analysis (2 h EdU pulse, Supplementary Fig. 2d–g) of Dot1L mutants and controls in the *Rosa26-Fucci2a* background. As left ventricle-specific transcripts were particularly affected in E16.5 Dot1L cKO hearts, we wondered whether a similar chamber tropism was operative for the neonatal proliferative phenotype. Quantification of chamber-specific rates of proliferation (as assessed by Fucci2a reporter, pH3 or EdU incorporation, Supplementary Fig. 6a, b, f) revealed similar results in all compartments analyzed (RV, septum, LV, RA and LA), suggesting that the cell cycle phenotype is mechanistically distinct from the decreased expression of patterning genes observed during embryogenesis.

**Fig. 7:**
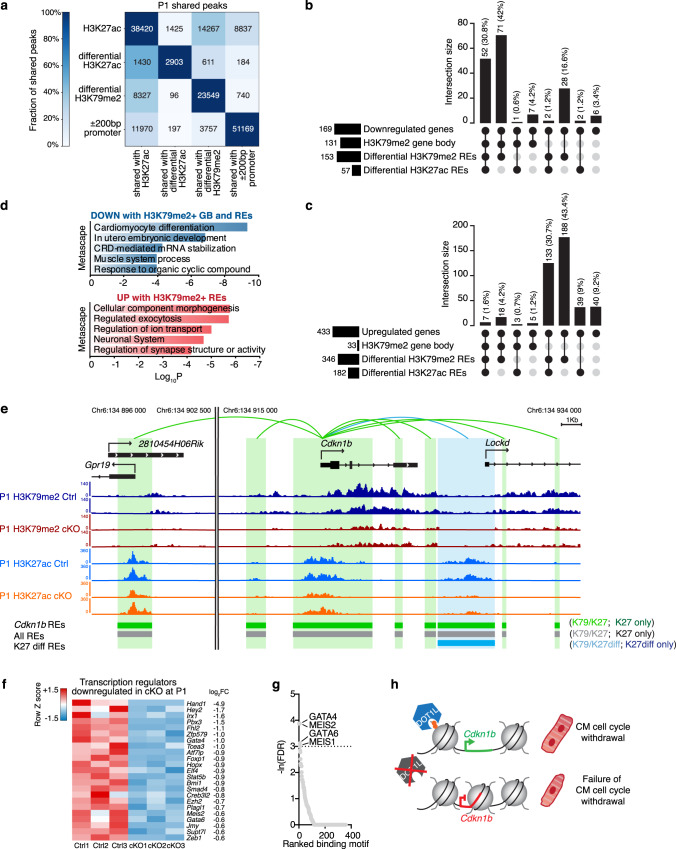
Mechanisms underlying sustained proliferation of DOT1L cKO cardiomyocytes. **a** Heatmap indicating the number of total and shared regions between differential H3K27ac ChIP-seq peaks, differential H3K79me2 ChIP-seq peaks and promoters (±200 bp around 5’TSS) in P1 CMs. Different intensities of colors indicate the fraction (%) of shared peaks. UpSet plots indicating the number and percentage of genes down- (**b**) and upregulated (**c**) in P1 Dot1L cKO versus Ctrl CMs with or without H3K79me2 in gene body (GB) and/or regulatory elements (REs) and with and/or without differential H3K27ac REs. **d** Metascape pathway analysis of downregulated genes with H3K79me2 in GB and K79-REs (top) and upregulated genes without gene body H3K79me2 but with K79-REs (bottom) in Dot1L cKO vs Ctrl P1 CMs. Top 5 enriched categories are shown, sorted by Log_10_ *P* value. **e** Browser tracks displaying H3K79me2 and H3K27ac ChIP-seq profiles of Ctrls (dark and light blue respectively) and Dot1L cKOs (red and orange respectively) P1 CMs in the genomic region harboring the *Cdkn1b* locus (encoding p27). Loops display all regulatory interactions between REs and the *Cdkn1b* gene, as identified by the ABC analysis. Gray loops identify interactions with REs without H3K79me2, green loops identify interactions with K79-REs, whereas blue loops represent interactions with K79-REs that additionally have H3K27ac REs differentially enriched between Dot1L Ctrls and cKOs. For reference, all REs are displayed in the middle lane in black or gray, regardless of their regulatory association with the *Cdkn1b* gene. Differential H3K27ac REs are indicated in the bottom lane in light blue when they overlap a K79-RE or dark blue when they do not. **f** Heatmap showing the expression of all transcription regulators downregulated in Dot1L cKO CMs at P1. **g** Motif enrichment analysis ranking transcription factors (TFs) enriched in REs associated with downregulated genes with H3K79me2 in GB and K79REs versus upregulated genes without H3K79me2 in GB and with K79REs. **h** Diagrammatic representation of mechanism of defective CM cell cycle withdrawal in the absence of DOT1L.

To gain insight into gene expression networks underlying the sustained proliferation of Dot1L cKO CMs, we used neonatal (P1) FACS-sorted CMs to perform mechanistic analyses similar to those described for E16.5: RNA-seq, H3K79me2 and H3K27ac ChIP-seq, followed by bioinformatic analysis leveraging a publicly available CM Hi-C dataset^31^ and employing the ABC method to predict interactions between regulatory elements and their target genes (Fig. 7, Supplementary Fig. 7 and Supplementary Data 1b–4b and 6b). These analyses revealed that, similar to E16.5, the majority of genes downregulated in cKO CMs (72.8%) were directly regulated by DOT1L via H3K79me2 both in gene body and REs (Fig. 7b). On the other hand, the vast majority of genes upregulated in cKOs (92.4%) did not have gene body H3K79me2, but 79.9% had interactions with K79-REs (Fig. 7c). Similar to what we had observed in E16.5 CMs, from all K79-REs, the vast majority (96%) did not show altered H3K27ac signal in Dot1L cKO CMs (Supplementary Data 3b). Differential H3K27ac was not a requirement for gene expression regulation by K79-REs as, within differentially expressed genes associated with K79-REs, 65% of downregulated genes and 60% of upregulated genes did not show differences in H3K27ac between genotype groups (Fig. 7b, c and Supplementary Data 4b). Functional annotation revealed that multiple genes upregulated in cKOs and associated with K79-REs had important roles in the neuronal system and in synaptic structure or activity. qPCR analyses (Supplementary Fig. 8) showed that genes in these categories were already upregulated in Dot1L cKO hearts in early cardiogenesis (E10.5), suggesting that their modulation is a direct consequence of DOT1L absence, rather than an indirect effect. On the other hand, genes downregulated in cKOs with H3K79me2 in gene body and REs were involved in CM differentiation (Fig. 7d and Supplementary Data 5b). Within these was the gene encoding the cell cycle regulator p27 (*Cdkn1b*) (Fig. 7e). Interestingly, p27 knockouts have a phenotype of increased heart size due to defective CM cell cycle arrest that resembles the one of Dot1L cKOs^35^. This observation strongly suggested that disrupted p27 expression accounts, at least in part, for the sustained CM proliferation observed in Dot1L cKOs (Fig. 7h). In addition to p27, it is likely that this phenotype is also mediated by other upstream factors. To identify other factors potentially involved in the sustained proliferation observed in Dot1L cKOs, we determined transcriptional regulators downregulated in Dot1L cKOs (Fig. 7f) and assessed transcription factor binding sites enriched in the K79-REs associated with genes downregulated in Dot1L cKOs with H3K79me2 in their gene body and in K79-REs (Fig. 7g). Together, these analyses suggested that TFs such as MEIS1 and MEIS2 might also play a role in the observed proliferative phenotype, which is consistent with known functions for these TFs in CM development post birth^36,37^. Altogether these observations suggested that in the neonatal period DOT1L directly promotes expression of genes involved in CM maturation and cell cycle withdrawal whilst repressing expression of genes associated with non-CM functions.

## Discussion

Devising a blueprint for transcriptional and epigenetic mechanisms regulating normal heart development is of critical importance for our understanding of congenital heart disease and to pave the way for regenerative therapies of the adult heart post injury. Our previous studies using embryonic stem cells led to the hypothesis that DOT1L might play a relevant role in cardiogenesis, which we confirmed with the in vivo experiments reported here. In addition, we deciphered previously unrecognized mechanisms of action of this epigenetic modifier. *Dot1l* is ubiquitously expressed in the heart and its CM-specific conditional ablation resulted in a fully penetrant phenotype of an enlarged and rounded heart that culminated in neonatal lethality (Figs. 1 and 2). Genome-wide transcriptomic and ChIP-seq assays provided detailed mechanistic insight into gene expression programs underlying this phenotype, highlighting DOT1L as a direct regulator of two processes that take place at distinct developmental stages: expression of critical cardiogenic factors in embryogenesis (Figs. 3, 4 and 5) and neonatal CM maturation and cell cycle withdrawal (Figs. 6 and 7). Detailed bioinformatic analyses revealed that DOT1L regulated expression of its targets via methylation of H3K79 in gene body, regulatory regions, or a combination of both. Changes in H3K27ac profiles were observed in a significant number of K79-REs associated with genes differently expressed in Dot1L cKOs, but were not a requirement for gene expression regulation by K79-REs.

In embryogenic stages, DOT1L directly regulated several genes involved in defining distinct cardiac chambers. Amongst these, there was a clear enrichment in genes required for left ventricular identity (*Hand1*, *Tbx5*, *Cited1*, *Gja5*). From these, *Hand1* emerged as a gene particularly sensitive to absence of DOT1L activity. Likely reflecting this tight regulation, the dilated and rounded heart phenotype with neonatal lethality displayed by Dot1L mutants strongly resembles the phenotype of *Hand1* conditional mutants^21^. How disruption of a left ventricle-specific transcription factor results in a rounded heart morphology is not currently clear and addressing this question will require a full understanding of gene expression networks downstream of HAND1. *Hand1* cardiac cKOs also display ventricular septal and outflow defects, whereas Dot1L mutants do not. This is likely a consequence of residual *Hand1* transcripts at earlier embryonic stages – *Hand1* transcripts were completely absent from E16.5 Dot1L cKO CMs but could be detected at E10.5 (although at much lower levels than in stage-matched controls, Fig. 3). Interestingly, postnatally *Hand1* is restricted to the cardiac conduction system and disrupting this specific expression causes conduction system problems^18^. Therefore, it is conceivable that electrocardiographic anomalies observed in Dot1L cKOs are, at least in part, caused by blunted *Hand1* expression. The dependence of *Hand1* expression on DOT1L is of particular relevance for our understanding of epigenetic regulation of cardiogenesis. Previous studies have shown that SMYD1 (that catalyzes H3K4 methylation) is critical for activation of *Hand2*^22^, but an epigenetic mechanism specifically regulating *Hand1* has remained elusive. Together with these previous observations, our results suggest a model in which DOT1L and SMYD1, two enzymes expressed throughout the heart, are essential for maintenance of normal levels of asymmetrically expressed cardiogenic factors (Fig. 5g).

DOT1L depletion resulted in absence of *Hand1* expression without affecting expression of *Hand2*, whereas absence of SMYD1 results in blunted activation of *Hand2* without affecting expression of *Hand1*^22^. DOT1L also regulated expression of *Smyd1* itself, revealing a complex epigenetic mechanism controlling normal cardiogenesis. It is unclear if SMYD1 is involved in regulating *Dot1l* expression. Interestingly, despite the fact that *Smyd1* transcripts were significantly downregulated in Dot1L cKOs, its critical target *Hand2* was not altered in these mutants, suggesting that the levels of SMYD1 protein present in Dot1L cKOs are sufficient to sustain mechanisms of transcriptional regulation dependent on this enzyme. Despite regulating distinct *Hand* genes, DOT1L and SMYD1 also share some common targets. For example, transcription of *Irx4* is dependent on the action of both enzymes (our own data and ref. 22). IRX4 is required for ventricular myocyte identity, and it is conceivable that DOT1L and SMYD1 act in a coordinated way to ensure a rheostatic control of *Irx4* transcription. How DOT1L and SMYD1, two enzymes that are expressed throughout the heart, achieve regulation of chamber-specific genes (ventricular versus atrial, left versus right) is not currently known and will be the subject of future studies. Given that several of DOT1L targets are also targeted by critical regulators of cardiogenesis, such as NKX2.5 and TBX5, it is possible that the specificity of these epigenetic enzymes is derived from their interactions with distinct transcription factors.

Initial studies suggested that H3K79 methylation is a generic marker of active transcription^5^, but our datasets clearly revealed that, in CMs, H3K79me2 methylation is not a basic requirement for gene transcription, as in control animals, multiple genes are active without having gene body H3K79 methylation. From the group of genes that bear this modification in controls and lose it in mutant hearts, two major categories emerge: those that are modulated upon DOT1L loss and those that are unaffected by the absence of this enzyme. The latter may reflect mechanisms of epigenetic redundancy. Our bioinformatic analyses integrating differential H3K79me2 ChIP-seq peaks with a mark of regulatory regions^38^ (CM H3K27ac ChIP-seq) and 3-dimensional genomic conformation (CM Hi-C) suggested a model in which, in addition to gene body H3K79me2, DOT1L mediates expression of target genes via H3K79me2 in cis-regulatory elements. In loci with gene body H3K79me2, those associated with K79-REs were more downregulated in Dot1L cKOs. The magnitude of downregulation correlated with two variables: extent of H3K79me2 gene body coverage and the number of K79-REs interacting with the gene, suggesting these two forms of H3K79me2 synergize to promote expression of target genes. Interestingly, our analyses suggested that K79-REs could also be of an inhibitory nature, blocking transcription of target genes that lack gene body H3K79me2. To our knowledge this is the first report of an involvement of DOT1L/H3K79me2 in this form of gene expression regulation, however, it is also conceivable that gene upregulation observed in Dot1L cKOs reflects secondary effects (for example, downregulation of a transcription regulator with repressor properties).

In mice, after the first week of life, most CMs are binucleated and completely withdrawn from the cell cycle^23^. Resistance of mature CM to proliferation is a major hurdle for cardiac regeneration^39,40^. Thus, devising strategies to promote reacquisition of proliferative potential in CMs is a major priority in cardiac regenerative medicine^39,40^. FACS and histological studies using biochemical and genetic strategies to identify CM-specific proliferation revealed that Dot1L cKO CMs retain proliferative potential at P10, a stage at which control counterparts are already withdrawn from the cell cycle (Fig. 6). Notably, Dot1L cKO hearts also had a higher percentage of mononucleated CMs than control hearts. Transcriptome analyses suggested that DOT1L depleted CMs are less mature than control CMs and fail to activate expression of p27, a known repressor of cell cycle. Permanent ablation of DOT1L results in severe consequences and lethality, but our results revealing sustained proliferation of DOT1L-depleted CMs suggest that temporary inhibition of DOT1L in postnatal hearts might be a strategy to promote re-acquisition of mitotic potential in CMs. However, this sort of approach would need to be finely titrated in follow up studies, as there are potential risks associated with the activation of genes normally repressed in CMs or downregulation of DOT1L-dependent genes necessary for normal CM function.

## Methods

### Mouse strains and experiments

Animal experiments were conducted according to protocols approved by the Institutional Animal Care and Use Committee at University of California, San Diego and the German local ethic committee (Regierungspräsidium Darmstadt, Hessen). All transgenic lines used were kept on an outbred background (Mus Musculus, Black-Swiss, Charles River laboratories). Mice were maintained in plastic cages with filtered air intake ports (Techniplast) on a 12 h light cycle and have free access to water and food (Teklad LM-485 irradiated diet, Harlan Laboratories, catalog number 7912). All mouse housing rooms are maintained at 72 +/−2 degrees Fahrenheit and 30–70% relative humidity. Adult (2–12 months old) males and females were used for breeding. For analyses conducted in embryonic stages, embryos were staged according to the embryonic day (E) on which dissection took place, with noon of the vaginal plug day being considered as E0.5 and birth typically occurring at E19. Experimental mice (males and females) were analyzed from embryonic day E10.5 to postnatal day 10. *Dot1l*^flox^ mice were obtained from the KOMP Repository (CSD29070) https://www.komp.org/geneinfo.php?geneid=54455. Floxed-out *Dot1l*^*Δ*^ mice were generated by crossing the *Dot1l*^flox^ allele with the epiblastic *Meox2-Cre* allele obtained from JAX laboratories (Stock No: 026858). *xMlc2-Cre* mice^24^ were gently provided by Timothy Mohun. The *Rosa26-tdTomato (Ai9) (tdTom*) indicator allele^25^ was purchased from JAX (Stock No: 007905). The Rosa26-*Fucci2A* cell cycle reporter allele was gently provided by Ian James Jackson^34^. All experiments were performed using littermate cKOs and Ctrls. Images shown are representative examples of experiments with *n* ≥ 3 biological replicates. In experiments assessing proliferation, mice received an injection of EdU (at P1 25 μL of a 3 g/L stock and at P10 50 μL of a 3 g/L stock) 2 h or 24 h prior to euthanasia.

For genotyping, genomic DNA was extracted by adding 250 μL of 50 mM NaOH to a tail tip biopsy and heating at 95 °C for 30 min. The solution was then neutralized by adding 50 μL of 1 M Trish-HCl (pH 8.0). Genotyping PCR reactions (36 amplification cycles) were performed using Taq DNA Polymerase with ThermoPol Buffer (New England Biolabs, M0267L), dNTPs from Promega (U1511) and 1 μL of DNA solution. The following genotyping primers were used:

XMlc2Cre-Fw 5’-TAGGATGCTGAGAATCAAAATGT-3’;

XMlc2Cre-Rev 5’-TCCCTGAACATGTCCATCAGGTTC-3’;

Dot1L-Fw 5’-CCATATTAGTGTTCAAGGGCTACT-3’;

Dot1L-fl/wt-Rev 5’-AGCATAAGGATGCCAACTACTAAC;

Dot1L-null-Rev 5’-AAGGAGGTCCTACTCATAGTCCTT-3’;

Rosa26-tdTomato-Fw 5’-CTGTTCCTGTACGGCATGG-3’;

Rosa26-tdTomato-Rev 5’-GGCATTAAAGCAGCGTATCC-3’;

Rosa26-wt-Fw 5’-AAGGGAGCTGCAGTGGAGTA-3’;

Rosa26-wt-Rev 5’-CCGAAAATCTGTGGGAAGTC-3’;

Rosa26-wt-Fw 5’-CAAAGTCGCTCTGAGTTGTTATCAG-3’;

Rosa26-wt-Rev 5’-GGAGCGGGAGAAATGGATATGAAG-3’;

Rosa26-Fucci2a-Rev 5’-TCACCCAGGAGTCATTTGAT-3’.

### Echocardiography and electrocardiography

Echocardiography was performed at P5 and P10 using a Vevo3100 imaging system (VisualSonics) with MX550 (for P10) or MX700 (for P5) probes. Two-dimensional-guided M-mode images of the short axis at the papillary muscle level were recorded. Data were analyzed by VevoLab 3.2.5 software using Auto-LV technology to minimize the variability between the measurement and confirmed by an experienced researcher. For electrocardiography (ECG) measurements the PR interval was measured from the beginning of the P-wave to the beginning of the QRS complex; QRS duration was measured from the first deflection of the Q-wave to the nadir of the S-wave (defined as the point of minimum voltage in the terminal portion of the QRS complex); the R-R interval was obtained as the average R-R interval over the sampling period.

### Immunohistochemistry

Tissues were isolated in cold PBS and fixed in 4% paraformaldehyde at 4 °C overnight. Tissues were dehydrated in a sucrose gradient (5% for 1 h, 12% for 1 h, 20% overnight) and embedded in a 1:1 mix of 20% Sucrose and Tissue-Tek Optimal Cutting Temperature compound (OCT, Sakura). 10 μm thick histological sections were cut using a cryostat (Leica CM3050) and kept at −20 °C (short-term) or −80 °C (long-term) until being processed for immunofluorescence. Sections or isolated CMs were blocked in 10% donkey serum before incubation with antibodies. Primary antibodies were incubated at 4 °C overnight. The following primary antibodies were used: anti-α Sarcomeric Actinin (1:400 Abcam #ab68167), anti-Myomesin (1:200 mMaC #B4 developed by Perriard, J.-C. was obtained from the Developmental Studies Hybridoma Bank, created by the NICHD of the NIH and maintained at The University of Iowa), anti-Vimentin (1:100 Abcam #ab45939), anti-TNNT (1:100 Thermo Fisher #MA5-12960), anti-PDGFR-α (1:200 R&D Systems #AF1062), anti-Collagen 1 (1:200 Abcam #ab34710), anti-tdTomato (1:100 Sicgen #ab8181-200), anti-GFP (1:600 Abcam #ab13970), anti-phosphoH3 (1:100 Millipore #06-570). EdU incorporation was detected using a Click-iT® EdU kit (Thermo Fisher Scientific #C10340). Secondary antibodies were incubated at room temperature for 1 h and 30 min. The following secondary antibodies were used at a concentration of 1:400: donkey anti-rabbit Alexa fluor 647 (Thermofisher #A31573), donkey anti-goat Alexa fluor 488 (Thermofisher #A11055), donkey anti-goat Alexa fluor 555 (Thermofisher #A21432), donkey anti-chicken Alexa fluor 488 (Jackson Immuno Research #703-545-155), donkey anti-rabbit Alexa fluor 555 (Thermofisher #A31572), donkey anti-mouse Alexa fluor 488 (Thermofisher #A21202), donkey anti-goat Alexa fluor 647 (Thermofisher #A21447). Masson Trichrome staining of tissue sections was performed using the Masson trichrome stain kit (Sigma, HT15), following instructions provided by the manufacturer. Immunofluorescence images were acquired using a Keyence BZX-700 fluorescent microscope, an Olympus FV1000 or a Leica SP8 scan confocal microscope. Bright field images were acquired using a Nikon Eclipse C1 microscope. Image processing was performed with Fiji and Volocity software.

### RNA scope

RNAscope fluorescent in situ hybridizations (ISH) were conducted using the RNA-scope Multiplex Fluorescent Reagent Kit v.2 (Advanced Cell Diagnostics #323100) following standard protocols provided by the manufacturer, using the following RNA-scope probes (ACDbio): Mm-Dot1L-C2 (#533431-C2); Mm-Hand1-C2 (#429651-C2); Mm-Irx4 (#504831); Mm-Hand2 (#499821); Mm-Smyd1-C3 (#1152831-C3).

### Cardiomyocyte isolation

Embryonic and neonatal CMs were isolated using a modified version of the a previously described protocol^41^. Embryonic or postnatal day 1 hearts were isolated and transferred into ice cold HBSS. Embryonic single cell suspensions were obtained by performing eight rounds of enzymatic digestion (5 min each) with Collagenase type II (0.7 mg/ml Worthington) at 37 °C under agitation. Postnatal day 1 single cell suspensions were obtained by performing an overnight digestion with trypsin (0.5 mg/ml) at 4 °C followed by eight rounds of enzymatic digestion (5 min each) with Collagenase type II (1 mg/ml Worthington) at 37 °C. Cells were collected in cold medium containing 10% horse serum and 5% fetal bovine serum to stop the enzymatic reaction and centrifuged at 210 rcf to allow initial separation of CMs from other cardiac cells. Cell preps were resuspended in FACS buffer (HBSS, 5%FBS, 2.5 mM EDTA) and incubated for 30 min with APC-conjugated, FACS-validated antibodies against CD31 (endothelial cells, clone: MEC13.3; BioLegend #102510), CD45 (leukocytes, clone: 30-F11, BioLegend #103112), CD140a (fibroblasts, clone: APA5, eBioscience #17-1401-81) and TER119 (erythroid cells, clone: TER-119, BioLegend #116212) to avoid doublets between CMs and stromal cells. Live/Dead (Invitrogen #L34957) or DAPI staining was performed to exclude dead cells. Embryonic and postnatal day 1 live and single control and mutant CMs were sorted based on the red fluorescence emitted by the Cre reporter tdTomato using a FACS Aria II or Influx Cell sorter (BD Biosciences) and collected in TRIZol reagent for RNA extraction or cross-linked as described below for chromatin analysis.

Postnatal day 10 CMs were isolated using a Langendorff system using Collagenase type II (0.7 mg/ml Worthington). After perfusion cells were dissociated from the heart, collected in conical tubes and allowed to settle by gravity in order to obtain separation of viable, rod-shaped CMs from dead CMs and from interstitial cells of the heart. For histological analysis, isolated CMs were fixed in 4%PFA and processed for immunostaining.

### Protein isolation and Western Blot analysis

Total protein extracts were prepared by lysing samples in RIPA buffer. Protein lysates in Laemmli buffer were separated by electrophoresis on 12% SDS-PAGE gels and transferred for 2 h at 4 °C on to a PVDF membrane (BioRad). After blocking for an hour in 5% dry milk, membranes were incubated overnight at 4 °C with the primary antibody in blocking buffer. The following primary antibodies were used: H3K79me2 (1:1000 abcam #ab3594) and anti-H3 (1:5000 abcam #ab1791). Blots were washed and incubated with a horseradish peroxidase (HRP)-conjugated secondary antibody generated in Rabbit (1:2000; Cell Signaling Technology #7074) for 1.5 h at room temperature. Immunoreactive protein bands were visualized using an enhanced chemiluminescence reagent (Thermo Fisher Scientific). Protein quantification was achieved using ImageJ software.

### Quantification of proliferation by Flow Cytometry

FACS quantification of rates of EdU incorporation was carried out using littermate Dot1L cKOs and Ctrls. EdU detection was done in cell suspensions using the Click-iT® EdU Alexa 647 kit (Thermo Fisher Scientific; C10340), according to the manufacturer’s instructions. TdTomato signal was used to discriminate CMs from other lineages of cardiac cells. Stained cells were analyzed using a FACS Canto II flow cytometer (BD Bioscience). DIVA (v8.0.1) and FlowJo (v10.8.1) software (BD Pharmingen) were used for data acquisition and analysis.

### RNA extraction, qRT-PCR and RNA-seq

RNA was extracted using Tryzol (Invitrogen #15596026) and Direct-zol RNA Kits (Zymo Research #R2061) following instructions provided by the manufacturers. All transcriptomics analyses (qRT-PCR or RNA-seq) were performed on FACS-sorted CMs, except qPCR in Supplementary Figs. 3a and 8 that were performed on whole heart tissue. For E16.5, P1, P5 and P10 biological replicates were prepared from single hearts. For E12.5, preparation of each biological replicate required pooling of hearts with same genotypes. For each stage analyzed, a minimum of 3 biological replicates, prepared from littermate Dot1L Ctrl and Dot1L cKO hearts, were used. For qRT-PCR experiments, cDNA was produced using the SuperScript VILO cDNA Synthesis Kit (Invitrogen #11754050). qRT-PCR was performed using SYBR Select Master Mix for CFX (Applied biosystems #4472952) on a Bio-Rad CFX96 Real-Time PCR system or Perfecta SYBR green FastMix (VWR # 733-1389) on an Applied Biosystems ViiA7 Real-Time PCR. The following primers were used:

Dot1L-Ex2-Fw 5’-TGCTGCTCATGAGATTATTGAGA-3’ (primer hybridizing within the loxP-flanked exon2 of the floxed *Dot1l* allele);

Dot1L-Ex4-Rev 5’-ATGGCCCGGTTGTATTTGTC-3’;

18s-Fw 5’-AAATCAGTTATGGTTCCTTTGGTC-3’;

18s-Rev 5’-GCTCTAGAATTACCACAGTTATCCAA-3’;

Col1a1-Fw 5’-CATGTTCAGCTTTGTGGACCT-3’;

Col1a1-Rev 5’-GCAGCTGACTTCAGGGATGT-3’;

Col3a1-Fw 5’-ACGTAGATGAATTGGGATGCAG-3’;

Col3a1-Rev 5’-GGGTTGGGGCAGTCTAGTG-3’.

Gap43-Fw 5’-ATAACTCCCCGTCCTCCAAGG-3’

Gap43-Rev 5’-GTTTGGCTTCGTCTACAGCGT-3’

Gpc2-Fw 5’-CTGCCCGGCATAGAAAGTTTA-3’

Gpc2-Rev 5’-GCGACCATAGGAATGCGAGAA-3’

Nefl-Fw 5’-TGATGTCTGCTCGCTCTTTC-3’

Nefl-Rev 5’-CTCATCCTTGGCAGCTTCTT-3’

Nefm-Fw 5’-ACAGCTCGGCTATGCTCAG-3’

Nefm-Rev 5’-CGGGACAGTTTGTAGTCGCC-3’

Rims4-Fw 5’-CTACTTCCCGTGCATGAACTC-3’

Rims4-Rev 5’-CCTCCATAGTTAAGGTTGCCCT-3’

Shank1-Fw 5’-TGCATCAGACGAAATGCCTAC-3’

Shank1-Rev 5’-AACAGTCCATAGTTCAGCACG-3’

Sybu-Fw 5’-GCGATGAAGACTTTACCAGGAA-3’

Sybu-Rev 5’-CCTCGGTTGCGTGAGAAAGA-3’

Ntng2-Fw 5’-GTGATGCGCCTGAAGGATTAT-3’

Ntng2-Rev 5’-TTCTCATGGGAACAGAACCTTTC-3’

Gabra4-Fw 5’-ACAATGAGACTCACCATAAGTGC-3’

Gabra4-Rev 5’-GGCCTTTGGTCCAGGTGTAG-3’

Gabrr1-Fw 5’-CGAGGAGCACACGACGATG-3’

Gabrr1-Rev 5-GTGAAGTCCATGTCAACCTCTG-3’

Snap25-Fw 5’- CAACTGGAACGCATTGAGGAA-3’

Snap25-Rev 5’-GGCCACTACTCCATCCTGATTAT-3’

Amph-Fw 5’-TCCGGGGATATTTAGCAGCAA-3’

Amph-Rev 5’-TGGCTCGTAGACTTCATGTAGAG-3’

Cadps2-Fw 5’-CTTGGTTGTCCGCTACGTTGA-3’

Cadps2-Rev 5’-GTTGAGCCATTGTTGACAGGC-3’

Erc2-Fw 5’-AAAGCAGCAGACCCAGAACA-3’

Erc2-Rev 5’-TGGTGGTGGTGGTAATGGTG-3’

Sh3gl2-Fw 5’-AACGATTGAATACCTCCAACCC-3’

Sh3gl2-Rev 5’-TTCACTTCCATGTCCAATGAGTC-3’

For RNA-seq experiments libraries were generated from 25 ng of RNA using the TruSeq Stranded mRNA library Prep kit (Illumina# 20020594) and sequenced on a HiSeq 4000 System (Illumina) using a single read 50 protocol.

### ChIP-seq

ChIP-seq was essentially performed as described^42,43^. Briefly, cells were fixed in in 1% formaldehyde/PBS for 10 min at room temperature, the reactions quenched by adding 2.625 M glycine to 125 mM final concentration, 20% BSA to 0.5% final concentration and cells pelleted by centrifugation for 5 min at 1000 g, 4 °C. Cells were washed twice with ice-cold 0.5% BSA/PBS and cell pellets were snap-frozen in liquid nitrogen and stored at −80 °C. For H3K79me2 ChIP-seq fixed cells were thawed on ice, resuspended in 500 µl ice-cold buffer L2 (0.5% Empigen BB, 1% SDS, 50 mM Tris/HCl pH 7.5, 1 mM EDTA, 1× protease inhibitor cocktail) and chromatin was sheared to an average DNA size of 300–500 bp by administering 7 pulses of 10 s duration at 13 W power output with 30 s pause on wet ice using a Misonix 3000 sonicator. The lysate was diluted 2.5-fold with ice-cold L2 dilution buffer (20 mM Tris/HCl pH 7.4@20 °C, 100 mM NaCl, 0.5% Triton X-100, 2 mM EDTA, 1× protease inhibitor cocktail), and one percent of the lysate was kept as ChIP input. For each immunoprecipitation, aliquots of diluted lysate equivalent to 150,000 to 1 million cells, 20 μl of Dynabeads Protein A (for rabbit polyclonal antibodies) and 2 μg anti H3K79me2 antibody (Abcam #ab3594) were combined and rotated overnight at 8 RPM and 4 °C. For H3K27ac ChIP-seq fixed cells were thawed on ice, resuspended in 100 µl ice-cold RIPA buffer (50 mM Tris-HCl pH 7.5, 150 mM NaCl, 1% IGEPAL CA-630 0.25% Na-Deoxycholate, 0.1% SDS, 1 mM EDTA, 1× protease inhibitor cocktail). Chromatin was sheared for 90 min on an Active Motif PIXUL high-throughput sonicator using standard settings (Pulse [N]: 50, PRF:1 kHz, Burst Rate: 20 Hz). Two microliter of each lysate was kept to generate ChIP input libraries, and the remainder used to immunoprecipitate H3K27ac-associated DNA by adding 2 μg anti-H3K27ac antibody (Active Motif 39133, lot 31521015) and 20 μl of Dynabeads Protein A (Thermo) and rotating overnight at 8 RPM and 4 °C. The following day, in the case of either H3K79me2 or H3K27ac ChIP-seq, beads were collected on a magnet and washed three times with 150 µl each of ice-cold wash buffer I (10 mM Tris/HCl pH 7.5, 150 mM NaCl, 1% Triton X-100, 0.1% SDS, 2 mM EDTA), wash buffer III (10 mM Tris/HCl pH 7.5, 250 mM LiCl, 1% IGEPAL CA-630, 0.7% Deoxycholate, 1 mM EDTA) and twice with ice-cold TET (10 mM Tris/HCl pH7.5, 1 mM EDTA, 0.2% Tween-20). Libraries were prepared directly on the antibody/chromatin-bound beads: after the last TET wash, beads were suspended in 25 μl TT (10 mM Tris/HCl pH7.5, 0.05% Tween-20), and libraries were prepared using NEBNext Ultra II reagents according to the manufacturer’s protocol but with reagent volumes reduced by half, using 1 µl of 0.625 µM Bioo Nextflex DNA adapters per ligation reaction. DNA was eluted, proteins digested and crosslinks reversed by adding 4 μl 10% SDS, 4.5 μl 5 M NaCl, 3 μl EDTA, 1 μl proteinase K (20 mg/ml), 20 μl water, incubating for 1 h at 55 °C, then overnight at 65 °C. DNA was cleaned up by adding 2 μl SpeedBeads 3 EDAC (Cytiva) in 61 μl of 20% PEG 8000/1.5 M NaCl, mixing and incubating for 10 minutes at room temperature. SpeedBeads were collected on a magnet, washed twice by adding 150 μl 80% EtOH for 30 s each, collecting beads and aspirating the supernatant. After air-drying the SpeedBeads, DNA was eluted in 25 μl TT and the DNA contained in the eluate was amplified for 12 cycles in 50 μl PCR reactions using NEBNext High-Fidelity 2X PCR Master Mix or NEBNext Ultra II PCR master mix and 0.5 μM each of primers Solexa 1GA and Solexa 1GB. Libraries were cleaned up as above by adding 36.5 μl 20% PEG 8000/2.5 M NaCl and 2 μl Speedbeads, two washes with 150 μl 80% EtOH for 30 s each, air-drying beads and eluting the DNA into 20 μl TT. ChIP library size distributions were estimated following 2% agarose/TBE gel electrophoresis of 2 μl library, and library DNA amounts measured using a Qubit HS dsDNA kit on a Qubit fluorometer. ChIP input material (1 percent of sheared DNA) was treated with RNase for 15 min at 37 °C in EB buffer (10 mM Tris pH 8, 0.5% SDS, 5 mM EDTA, 280 mM NaCl), then digested with Proteinase K for 1 h at 55 °C and crosslinks reversed at 65 °C for 30 min to overnight. DNA was cleaned up with 2 μl SpeedBeads 3 EDAC in 61 μl of 20% PEG 8000/1.5 M NaCl and washed with 80% ethanol as described above. DNA was eluted from the magnetic beads with 25 μl of TT and library prep and amplification were performed as described for ChIP samples. For H3K79me2 ChIP-seq, libraries were sequenced single-end for 75 cycles (SE75) to a depth of 20-25 million reads, for H3K27ac ChIP-seq libraries were sequenced single-end for 76 cycles (SE76) to a depth of 15-22 million reads on an Illumina NextSeq 500 instrument.

### Bioinformatic analyses

#### RNA-seq

Sequencing reads were processed to remove Illumina barcodes and aligned to the UCSC *Mus musculus* reference genome (build mm10) using STAR v.2.5.1b with default parameters^44^. ReadsPerGene.out.tab files were then processed with edgeR^45^. RNA expression was calculated in reads per kilobase per million mapped reads (RPKM) considering the sum of exon length. Differentially expressed coding genes were selected based on the following parameters: FDR ≤ 0.05, RPKM ≥ 1 in at least one biological condition, and log2 Fold Change ≤ −0.5 for genes downregulated and ≥0.5 for genes upregulated in Dot1L cKOs vs Dot1L Ctrls. Pathway analysis was performed using METASCAPE^46^.

#### ChIP-seq alignment and peak-calling

All analyses were performed using the mouse reference genome GRCm38 (mm10) and the gencode gene annotation version vM25. H3K79me2 and H3K27ac ChIP-seq data was processed in the same manner. Bowtie2^47^ was applied to align the fastq files to the mouse reference genome. First, the required index structure was built using: *bowtie2-build -f --seed 123 --threads 20 Mus_musculus.GRCm38.dna.primary_assembly.fa mouse_GRCM38_mm10*. To get the alignment files we ran: *bowtie2 -x mouse_GRCM38_mm10 -U*<*fastq-file*>*-S*<*output-file-name*>*.sam -q -t -p 30*. Conversion of the resulting files (sam) to bam format was done using samtools (version samtools 1.10)^48^. To allow easy visualization in a genome browser, bam files were converted to bigWig (bw) files using deeptools bamCoverage function. Next, peak-calling was performed with MACS2 (version macs2 2.2.7.1)^49^: *macs2 callpeak -t*<*treatment*>*.bam -c*<*input*>*.bam -n*<*prefix-name*>*--outdir*<*output-dir*>*-f BAM -g 1.87e9 -B*, where <treatment> is either the aligned reads of the Dot1L Ctrl or Dot1L cKO and <input> the corresponding input signal. For all following analyses we used the narrowPeak files. To compute differential ChIP-seq sites between Ctrl and Dot1L cKOs, we applied DiffBind (version 2.10.0 for H3K79me2 and version 3.4.11 for H3K27ac bioconductor-diffbind)^50^.

#### Determining genomic distribution of H3K79me2 or H3K27ac peaks

To determine the location of peaks in relation to distinct genomic regions, the gene annotation was split into exons, UTRs and gene bodies. Promoter regions of 400 bp length, centered around the most 5’ TSS, were added. All those genomic regions were made exclusive, so that each base pair of a gene had one unique label. The remaining base pairs located within gene bodies but not overlapping any other feature were labeled as introns. Based on those annotations, we determined the location of each base pair of each ChIP-seq peak, using bedtools (v2.25.0)^51^, and visualized their distribution with pie charts. ChIP-seq tracks were visualized with Integrative Genomics Viewer (IGV) (v2.9.2).

#### Computing coverage and fraction of the gene body covered by H3K79me2

The profiles of the H3K79me2 signal at the gene body were computed using deeptools^52^. We used the bam-files resulting from the bowtie2 analysis and the up- and downregulated genes in bed-file format (with strand information).

First, we applied deeptools bamCompare to determine the mean signal of the two replicates of Ctrl and cKO for each time point: *bamCompare -b1*<*treatment_rep1*>*.bam -b2*<*treatment_rep2*>*.bam -o*<*treatment*>*_mean_rep1_rep2.bw -of bigwig --scaleFactorsMethod None --operation mean --effectiveGenomeSize 2652783500 -p 25 --normalizeUsing RPKM --binSize 20 --skipNonCoveredRegions*, where <treatment> is either Ctrl or cKO.

Next, we visualized the data using deeptools computeMatrix and plotProfile functionalities:

*computeMatrix scale-regions -S Ctrl_mean_rep1_rep2.bw cKO_mean_rep1_rep2.bw -R downregulated_genes_FDR_0.05.bed upregulated_genes_FDR_0.05.bed -o inputProfile.mat.gz --endLabel TTS --beforeRegionStartLength 2000 --afterRegionStartLength 2000 --regionBodyLength 5000 -p 20 --skipZeros*

*plotProfile -m inputProfile.mat.gz -out profile.pdf --perGroup --startLabel TSS --endLabel TTS --plotTitle “Average distribution of H3K79me2 relative to the distance from TSS and TTS” --samplesLabel “Ctrl (upregulated genes)” “cKO (upregulated)” --regionsLabel “downregulated genes” “upregulated genes” --plotFileFormat pdf*

To compute the fraction of the gene body which is covered by H3K79m2, we applied bedtools (bedtools v2.25.0) coverage function. Given a set of genomic regions and a bam-file, the function computes the number of reads that overlap each genomic region and the fraction of bases that have a non-zero coverage based on the bam-file. We determined the number of overlapping reads and the fraction for all annotated mouse genes for Ctrl and cKO. Next, the mean fraction (of rep1 and rep2) of the up- and downregulated genes was computed. The density plots are based on all up- and downregulated genes.

#### Calling regulatory interactions with H3K27ac ChIP-seq and Hi-C

For the identification of enhancer regions H3K27ac ChIP-seq was performed on FACS sorted CMs at E16.5 and P1. A Hi-C matrix of mouse CMs was downloaded from GEO (GSM2544836)^31^. The Hi-C matrix was normalized using Knight-Ruiz normalization with the Juicebox dump command (version 1.22.01)^53^ with a resolution of 5000 bp for each chromosome.

*java -jar juicer_tools_1.22.01.jar dump observed KR*<*hic-file*>*chr$ chr$ BP 5000*<*output-file-name*>

The regulatory interactions between promoter and candidate enhancers were assessed with an adapted version of the ABC-score of Fulco et al.^27^ from the STARE software^28^. The command was as follows: *STARE_ABCpp -b*<*H3K27ac-peak-file*>*-n 7 -f*<*folder-with-HiC-files*>*-k 5000 -t 0.02 -a gencode.vM25.annotation.gtf -w 5000000 -o*<*output-path*>For all genes on the autosomes (GRCm38p6) the most 5’ TSS was taken and all candidate enhancers in a 5 Mb window centered on the TSS were scored according to the following equation:

1 $$\overline{ABC}scor{e}_{e,g}=\frac{{A}_{e,g}\cdot {C}_{e,g}}{{\sum }_{i\in {E}_{g}}{A}_{i,g}\cdot {C}_{i,g}}$$

The interaction of an enhancer (*e*) with a gene (*g*) is described by the gene-specific activity of the enhancer (*A*_*e,g*_) and the contact to the gene (*C*_*e,g*_). The adapted ABC-score returns the relative contribution of an interaction in relation to all other candidate interactions of that gene (*E*_*g*_). The gene-specific activity of an enhancer is defined as follows:

2 $${A}_{e,g}={A}_{e}\frac{{C}_{e,g}}{{\sum }_{j\in {G}_{e}}{C}_{e,j}}$$

where the activity of an enhancer (*A*_*e*_), measured as signal of the H3K27ac ChIP-seq peak, is taken relative to the contacts that an enhancer has to its candidate target genes (*G*_*e*_). The contact between an enhancer and a gene’s TSS is the contact frequency of the respective bins in the normalized Hi-C matrix. All candidate enhancer-gene interactions with an adapted score ≥ 0.02 were used for further analyses. Chromosomes X and Y were excluded from the analysis. Statistical differences between the cumulative log2(fold-change of gene expression) distributions were calculated pairwise with a two-tailed Kolmogorov-Smirnov test (SciPy 1.4.1, stats module, ks_2samp function).

#### Mapping differential ChIP-seq signal to genes

An RE was considered differential for H3K27ac/H3K79me2 if ≥10% of the enhancer’s length was covered by a differential ChIP-seq signal.

The REs differential for H3K27ac were mapped to genes via the adapted ABC-score. If a RE had a significant change in H3K27ac signal in cKO versus Ctrl, it was accounted for all genes that had an ABC interaction to that RE in Ctrl. Additionally, we considered H3K27ac signal changes in all REs present only in cKO that were mapped to a gene via ABC interactions in cKOs but not in Ctrls.

#### Motif enrichment analysis

To identify transcription factors likely regulating the activity of K79-REs associated to genes differentially expressed in Dot1L cKOs, a motif enrichment analysis was performed. To this end, K79-REs associated to differentially expressed genes were divided into two groups: those associated with genes downregulated in Dot1L cKOs with H3K79me2 in their gene body (activating K79-REs) and those associated with genes upregulated in Dot1L cKOs without H3K79me2 in their gene body (silencing K79-REs). TF binding motifs (total of 515) were downloaded from the JASPAR database^54^ and only those significantly expressed (RPKM ≥ 1) in E16.5 or P1 CMs were considered in subsequent analyses. Using TRAP^55^, a TF-affinity value per TF for each RE sequence was computed. The value is defined as the sum over all binding site probabilities of the given TF for the current sequence. The TRAP analysis was performed separately for the RE sequences of the up- and downregulated genes. A one-tailed Mann-Whitney test (using R’s Wilcox test function, confidence level of 0.975) to identify TFs enriched in the activating K79-REs versus silencing K79-REs and vice versa was performed. The resulting p-values were adjusted by applying the Benjamini–Hochberg procedure^56^. All TFs with an adjusted p-value of ≤ 0.05 were considered significant.

#### Pathway enrichment analysis

Pathway enrichment analysis of genes downregulated with H3K79me2 in gene body and regulatory elements and genes upregulated with H3K79me2 in regulatory elements but not H3K79me2 in gene body was performed using Metascape web tool^46^ using standard settings.

#### Quantification of experiments, statistical analysis and reproducibility

CM cell length and width and RNA-scope puncta were measured using Image J. Statistical significance of differences in the survival of DOT1L mice was assessed using Kaplan–Meier survival analysis with the log-rank method of statistics. In Fig. 2 graphs data are expressed as mean ± SD, in all other graphs, data are expressed as mean ± SEM, with a minimum of 3 biological replicates (the exact replicate number is described in the legend to each Fig.). Statistical significance of differences among groups was tested by 2-tailed Student’s *t* test. A value of *P* ≤ 0.05 was considered statistically significant. *represents *P* ≤ 0.05, **P ≤ 0.01. Analyses were performed with GraphPad Prism software. Each experiment was repeated independently with similar results at least 2–3 times.

### Reporting summary

Further information on research design is available in the Nature Portfolio Reporting Summary linked to this article.

## Supplementary information

Supplementary Information

Peer Review File

Description of Additional Supplementary Files

Supplementary Data 1

Supplementary Data 2

Supplementary Data 3

Supplementary Data 4

Supplementary Data 5

Supplementary Data 6

Reporting Summary

## Data Availability

All RNA-seq and ChIP-seq data that support the finding of this study have been deposited in the Gene Expression Omnibus (GEO) under accession number code GSE184192 Previously published Hi-C data that were re-analyzed here are available under accession codes GSM2544836 from GEO. Source data are provided with this paper.

## Source data

Source Data

## References

[1] Reik W (2007). Stability and flexibility of epigenetic gene regulation in mammalian development. Nature.

[2] Singer MS (1998). Identification of high-copy disruptors of telomeric silencing in Saccharomyces cerevisiae. Genetics.

[3] Feng Q (2002). Methylation of H3-lysine 79 is mediated by a new family of HMTases without a SET domain. Curr. Biol..

[4] McLean CM, Karemaker ID, van Leeuwen F (2014). The emerging roles of DOT1L in leukemia and normal development. Leukemia.

[5] Steger DJ (2008). DOT1L/KMT4 recruitment and H3K79 methylation are ubiquitously coupled with gene transcription in mammalian cells. Mol. Cell Biol..

[6] Godfrey L (2019). DOT1L inhibition reveals a distinct subset of enhancers dependent on H3K79 methylation. Nat. Commun..

[7] Cattaneo P (2016). DOT1L-mediated H3K79me2 modification critically regulates gene expression during cardiomyocyte differentiation. Cell Death Differ..

[8] Jones B (2008). The histone H3K79 methyltransferase Dot1L is essential for mammalian development and heterochromatin structure. PLoS Genet.

[9] Nguyen AT (2011). DOT1L regulates dystrophin expression and is critical for cardiac function. Genes Dev..

[10] Guimarães-Camboa N (2015). HIF1α represses cell stress pathways to allow proliferation of hypoxic fetal cardiomyocytes. Dev. Cell.

[11] Wu T (2022). PRDM16 is a compact myocardium-enriched transcription factor required to maintain compact myocardial cardiomyocyte identity in left ventricle. Circulation.

[12] Evans SM, Yelon D, Conlon FL, Kirby ML (2010). Myocardial lineage development. Circ. Res..

[13] Paige SL, Plonowska K, Xu A, Wu SM (2015). Molecular regulation of cardiomyocyte differentiation. Circ. Res..

[14] Olson EN (2006). Gene regulatory networks in the evolution and development of the heart. Science.

[15] Harvey RP (2002). Patterning the vertebrate heart. Nat. Rev. Genet.

[16] Srivastava D (2006). Making or breaking the heart: from lineage determination to morphogenesis. Cell.

[17] Bao ZZ, Bruneau BG, Seidman JG, Seidman CE, Cepko CL (1999). Regulation of chamber-specific gene expression in the developing heart by Irx4. Science.

[18] Vincentz JW (2019). Variation in a left ventricle-specific Hand1 enhancer impairs GATA transcription factor binding and disrupts conduction system development and function. Circ. Res..

[19] Srivastava D (1997). Regulation of cardiac mesodermal and neural crest development by the bHLH transcription factor, dHAND. Nat. Genet.

[20] Thomas T, Yamagishi H, Overbeek PA, Olson EN, Srivastava D (1998). The bHLH factors, dHAND and eHAND, specify pulmonary and systemic cardiac ventricles independent of left-right sidedness. Dev. Biol..

[21] McFadden DG (2005). The Hand1 and Hand2 transcription factors regulate expansion of the embryonic cardiac ventricles in a gene dosage-dependent manner. Development.

[22] Gottlieb PD (2002). Bop encodes a muscle-restricted protein containing MYND and SET domains and is essential for cardiac differentiation and morphogenesis. Nat. Genet.

[23] Soonpaa MH, Kim KK, Pajak L, Franklin M, Field LJ (1996). Cardiomyocyte DNA synthesis and binucleation during murine development. Am. J. Physiol..

[24] Breckenridge R, Kotecha S, Towers N, Bennett M, Mohun T (2007). Pan-myocardial expression of Cre recombinase throughout mouse development. Genesis.

[25] Madisen L (2010). A robust and high-throughput Cre reporting and characterization system for the whole mouse brain. Nat. Neurosci..

[26] Bruneau BG (1999). Chamber-specific cardiac expression of Tbx5 and heart defects in Holt-Oram syndrome. Dev. Biol..

[27] Fulco CP (2019). Activity-by-contact model of enhancer-promoter regulation from thousands of CRISPR perturbations. Nat. Genet.

[28] Hecker, D., Ardakani, F. B. & Schulz, M. H. The adapted Activity-By-Contact model for enhancer-gene assignment and its application to single-cell data. *bioRxiv*10.1101/2022.01.28.478202 (2022). 10.1093/bioinformatics/btad062 PMC9931646 36708003

[29] Li G (2012). Extensive promoter-centered chromatin interactions provide a topological basis for transcription regulation. Cell.

[30] Dao LTM (2017). Genome-wide characterization of mammalian promoters with distal enhancer functions. Nat. Genet.

[31] Rosa-Garrido M (2017). High-resolution mapping of chromatin conformation in cardiac myocytes reveals structural remodeling of the epigenome in heart failure. Circulation.

[32] Lai AY, Wade PA (2011). Cancer biology and NuRD: a multifaceted chromatin remodelling complex. Nat. Rev. Cancer.

[33] Bruneau BG (2001). A murine model of Holt-Oram syndrome defines roles of the T-box transcription factor Tbx5 in cardiogenesis and disease. Cell.

[34] Mort RL (2014). Fucci2a: a bicistronic cell cycle reporter that allows Cre mediated tissue specific expression in mice. Cell Cycle.

[35] Poolman RA, Li JM, Durand B, Brooks G (1999). Altered expression of cell cycle proteins and prolonged duration of cardiac myocyte hyperplasia in p27KIP1 knockout mice. Circ. Res..

[36] Mahmoud AI (2013). Meis1 regulates postnatal cardiomyocyte cell cycle arrest. Nature.

[37] Alam P (2019). Inhibition of senescence-associated genes Rb1 and Meis2 in adult cardiomyocytes results in cell cycle reentry and cardiac repair post-myocardial infarction. J. Am. Heart Assoc..

[38] Creyghton MP (2010). Histone H3K27ac separates active from poised enhancers and predicts developmental state. Proc. Natl Acad. Sci. USA.

[39] Guo Y, Pu WT (2020). Cardiomyocyte maturation: new phase in development. Circ. Res..

[40] Eschenhagen T (2017). Cardiomyocyte regeneration: a consensus statement. Circulation.

[41] Louch WE, Sheehan KA, Wolska BM (2011). Methods in cardiomyocyte isolation, culture, and gene transfer. J. Mol. Cell Cardiol..

[42] Heinz S (2018). Transcription elongation can affect genome 3D structure. Cell.

[43] Texari L (2021). An optimized protocol for rapid, sensitive and robust on-bead ChIP-seq from primary cells. STAR Protoc..

[44] Dobin A (2013). STAR: ultrafast universal RNA-seq aligner. Bioinformatics.

[45] Robinson MD, McCarthy DJ, Smyth GK (2010). edgeR: a bioconductor package for differential expression analysis of digital gene expression data. Bioinformatics.

[46] Zhou Y (2019). Metascape provides a biologist-oriented resource for the analysis of systems-level datasets. Nat. Commun..

[47] Langmead B, Salzberg SL (2012). Fast gapped-read alignment with Bowtie 2. Nat. Methods.

[48] Li H (2009). The sequence alignment/map format and SAMtools. Bioinformatics.

[49] Zhang Y (2008). Model-based analysis of ChIP-Seq (MACS). Genome Biol..

[50] Ross-Innes CS (2012). Differential oestrogen receptor binding is associated with clinical outcome in breast cancer. Nature.

[51] Quinlan AR, Hall IM (2010). BEDTools: a flexible suite of utilities for comparing genomic features. Bioinformatics.

[52] Ramírez F (2016). deepTools2: a next generation web server for deep-sequencing data analysis. Nucleic Acids Res..

[53] Durand NC (2016). Juicer provides a one-click system for analyzing loop-resolution Hi-C experiments. Cell Syst..

[54] Fornes O (2020). JASPAR 2020: update of the open-access database of transcription factor binding profiles. Nucleic Acids Res..

[55] Sobreira TJ, Durham AM, Gruber A (2006). TRAP: automated classification, quantification and annotation of tandemly repeated sequences. Bioinformatics.

[56] Benjamini Y, Hochberg Y (1995). Controlling the false discovery rate: a practical and powerful approach to multiple testing. J. R. Stat. Soc. Ser. B.

