# Supplementary material for: DOT1L regulates chamber-specific transcriptional networks during cardiogenesis and mediates postnatal cell cycle withdrawal
Source: Nat Commun. 2022 Dec 2;13:7444. doi: 10.1038/s41467-022-35070-2 (PMC9718823; doi:10.1038/s41467-022-35070-2)
Supplement: Supplementary file 1 — Supplementary Information [file 41467_2022_35070_MOESM1_ESM.pdf]

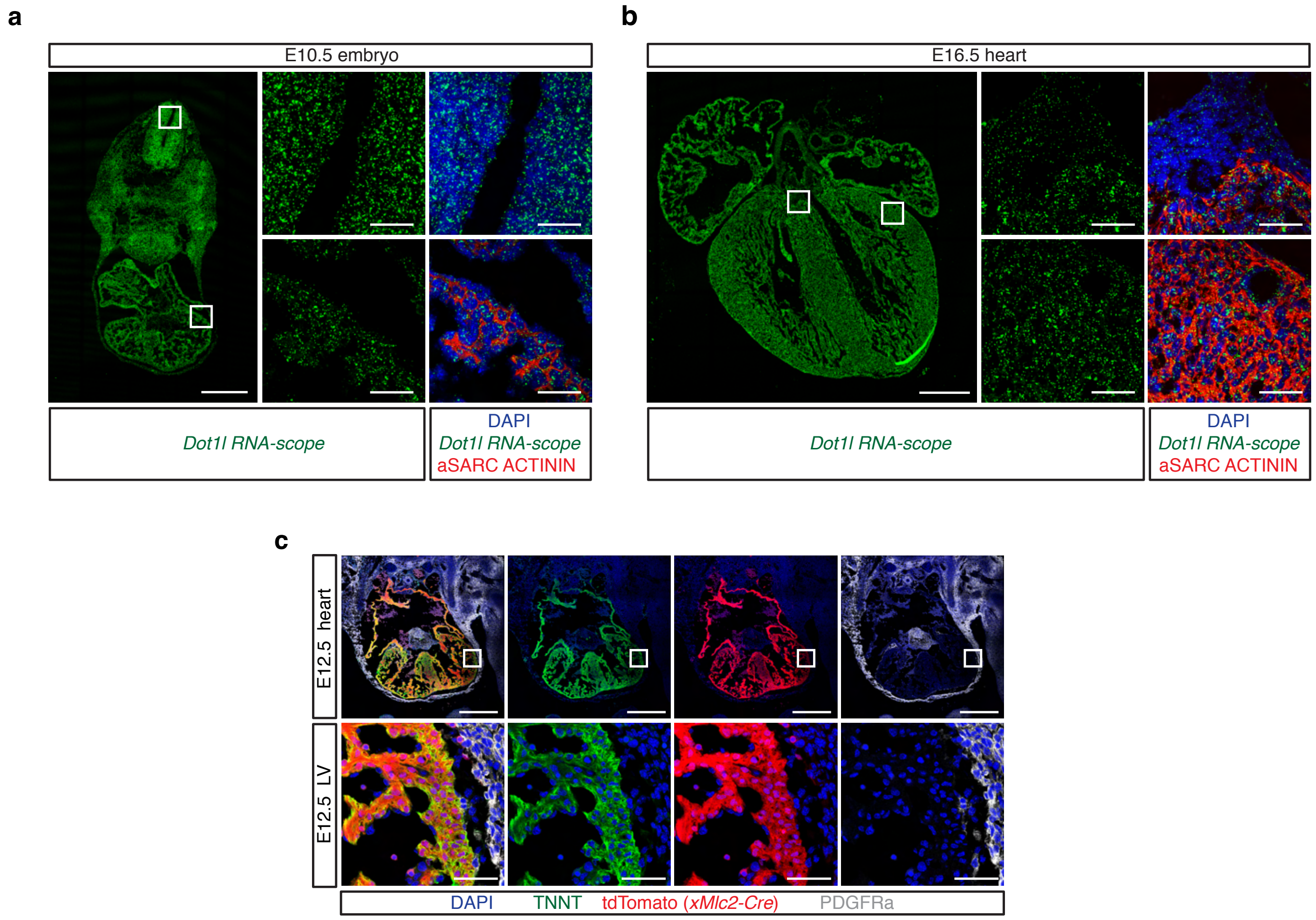

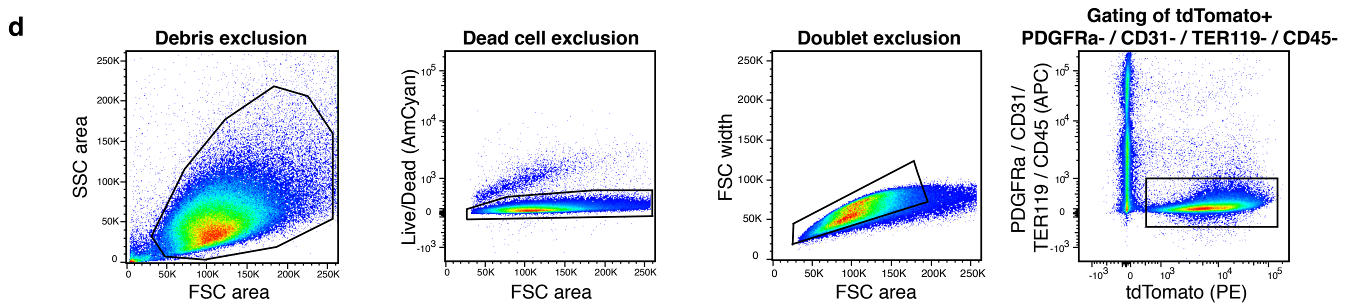

e

Stage	N litters	N mice	Without Cre $Dot1l^{fl/+}$	Without Cre $Dot1l^{null/fl}$	*xMlc2-Cre* $Dot1l^{fl/+}$	*xMlc2-Cre* $Dot1l^{null/fl}$
E12.5	23	190	30.0%	24.2%	21.6%	24.2%
E14.5	8	65	27.7%	13.8%	30.8%	27.7%
E16.5	30	255	24.7%	25.5%	25.9%	23.9%
E18.5	2	19	15.8%	10.5%	47.4%	26.3%
P0	33	261	25.3%	26.4%	27.2%	21.1%

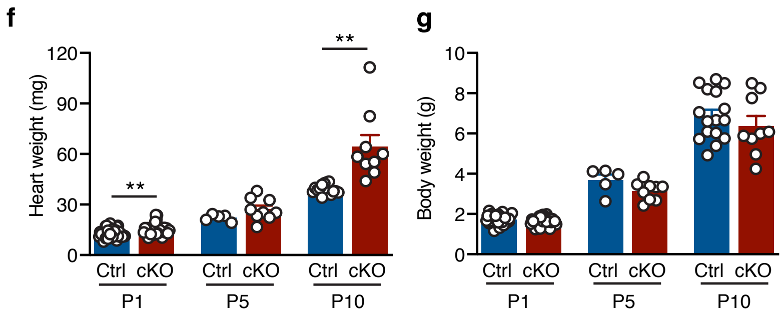

Supplementary Figure 1, related to Figure 1: Patterns of *Dot1l* expression and strategy for its conditional deletion in cardiomyocytes from early embryonic timepoints. a-b) RNA-scope detection of *Dot1l* transcripts in distinct embryonic stages. *Dot1l* was ubiquitously expressed in E10.5 embryos (a). In E16.5 heart, *Dot1l* mRNA could be detected in all cell types, but CMs (labeled by αSarcomeric Actinin in red) displayed higher abundance of *Dot1l* transcripts than non-myocyte

lineages (b) Scale bar = 500 μm for low magnification panels and 50 μm for high magnification of boxed areas). **c)** The *xMlc2-Cre* allele was highly specific and efficient in promoting recombination in embryonic CMs, as evidenced from the extensive overlap between the tdTomato recombination reporter signal (Cre+ cells, in red) and the CM marker Troponin T (TNNT, in green), as well as the lack of colocalization between tdTomato and the marker of mesenchymal lineages PDGFRα (white) (scale bar = 500 μm low magnification top panels; 50 μm for high magnification of boxed areas). **d)** FACS strategy to isolate highly pure populations of CMs based on the signal emitted by the red fluorescent protein tdTomato (expressed only in cells hit by the *xMlc2-Cre*). Negative selection for PDGFRα, CD31, TER119 and CD45 was used to exclude doublets with fibroblasts, endothelial cells, erythrocytes or leucocytes respectively. **e)** Observed genotype distribution of live embryos recovered from mating between *XMlc2-Cre;Dot1lΔ/+* and *Dot1lfl/fl* mice. No significant deviations from the expected Mendelian distribution (25% for each genotype) were observed until birth. **f)** Graph representing a significant increase in heart weight (mg) in Dot1L cKOs vs Ctrls in all stages analyzed. (P1 Ctrl N=36, P1 cKO N=30, P5 Ctrl N=5, P5 cKO N=9, P10 Ctrl N=16, P10 cKO N=9 biological replicates; unpaired t-test, two-sided P1 P= 0.0008, P10 P= <0.0001). **g)** No significant body weight differences were observed between Ctrl and cKO mice at P1, P5 and P10. (P1 Ctrl N=36, P1 cKO N=30, P5 Ctrl N=5, P5 cKO N=9, P10 Ctrl N=16, P10 cKO N=9 biological replicates). Data is presented as mean ± SEM; * represents P≤0.05, **P≤0.01. Source data are provided as a Source Data file.

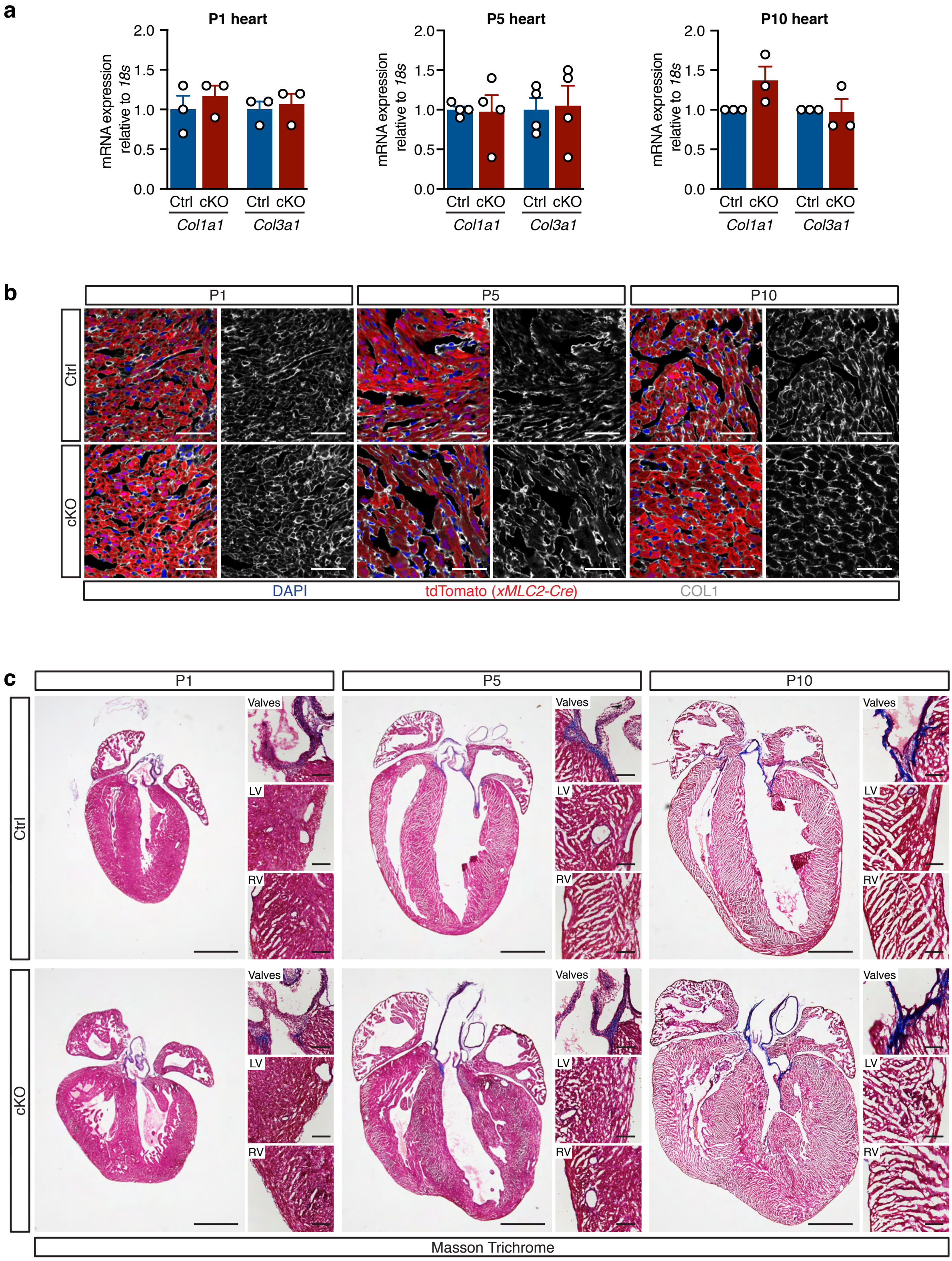

Supplementary Figure 2, related to Figure 1: Absence of fibrotic remodeling in Dot1L cKO hearts. a) qPCR for major Collagen genes *(Col1a1* and *Col3a1)* typically upregulated in fibrotic responses revealed absence of significant differences between Dot1L Ctrl and cKO whole hearts at all stages analyzed (P1, P5 and P10) (N=3 biological replicates, data are presented as mean ± SEM). **b**) Immunostaining for Collagen1 (COL1, white) showed comparable results in hearts from Ctrls and cKOs, DAPI in blue and endogenous tdTomato signal driven by *xMlc2*-Cre;Rosa26-tdTomato in red. (Scale bar = 50μm). **c**) Absence of fibrosis in Dot1L cKO vs Ctrl hearts was further confirmed by Masson trichrome staining at P1, P5 and P10. (Scale bar = 1 mm for low magnification panels and 100 μm for high magnification panels). Source data are provided as a Source Data file.

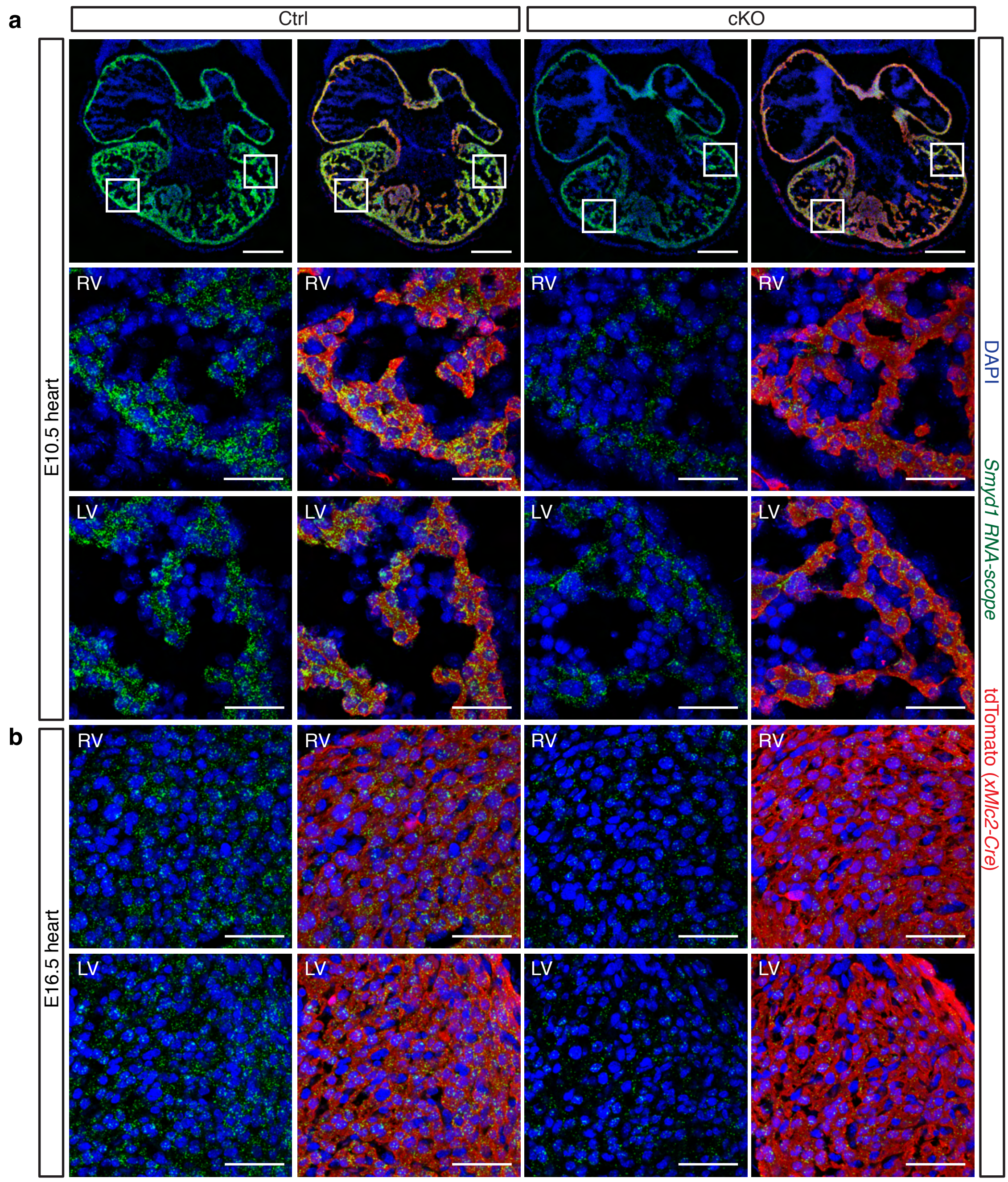

Supplementary Figure 3, related to Figure 3: Reduced *Smyd1* expression in Dot1L cKO hearts. **a**) *Smyd1* expression is CM-restricted and can be observed in all chambers of control E10.5 hearts. At this stage, *Smyd1* transcript levels were reduced both in the right and left ventricles of Dot1L cKO hearts, as shown in the higher magnification images of boxed areas (quantification data in Fig. 3i), confirming that DOT1L is necessary for normal *Smyd1* expression. **b)** A similar downregulation of *Smyd1* transcript abundance was observed in both ventricles of E16.5 Dot1L cKO hearts. DAPI in blue, RNA-scope signal of *Smyd1* probe in green and tdTomato signal driven by *xMlc2*-Cre;Rosa26-tdTomato in red. To allow clear identification of RNA-scope signals, all images are shown both as a 2-color (*Smyd1* and DAPI) and 3-color merge. (Scale bar = 250 μm for low magnification panels and 50 μm for high magnification of boxed areas).

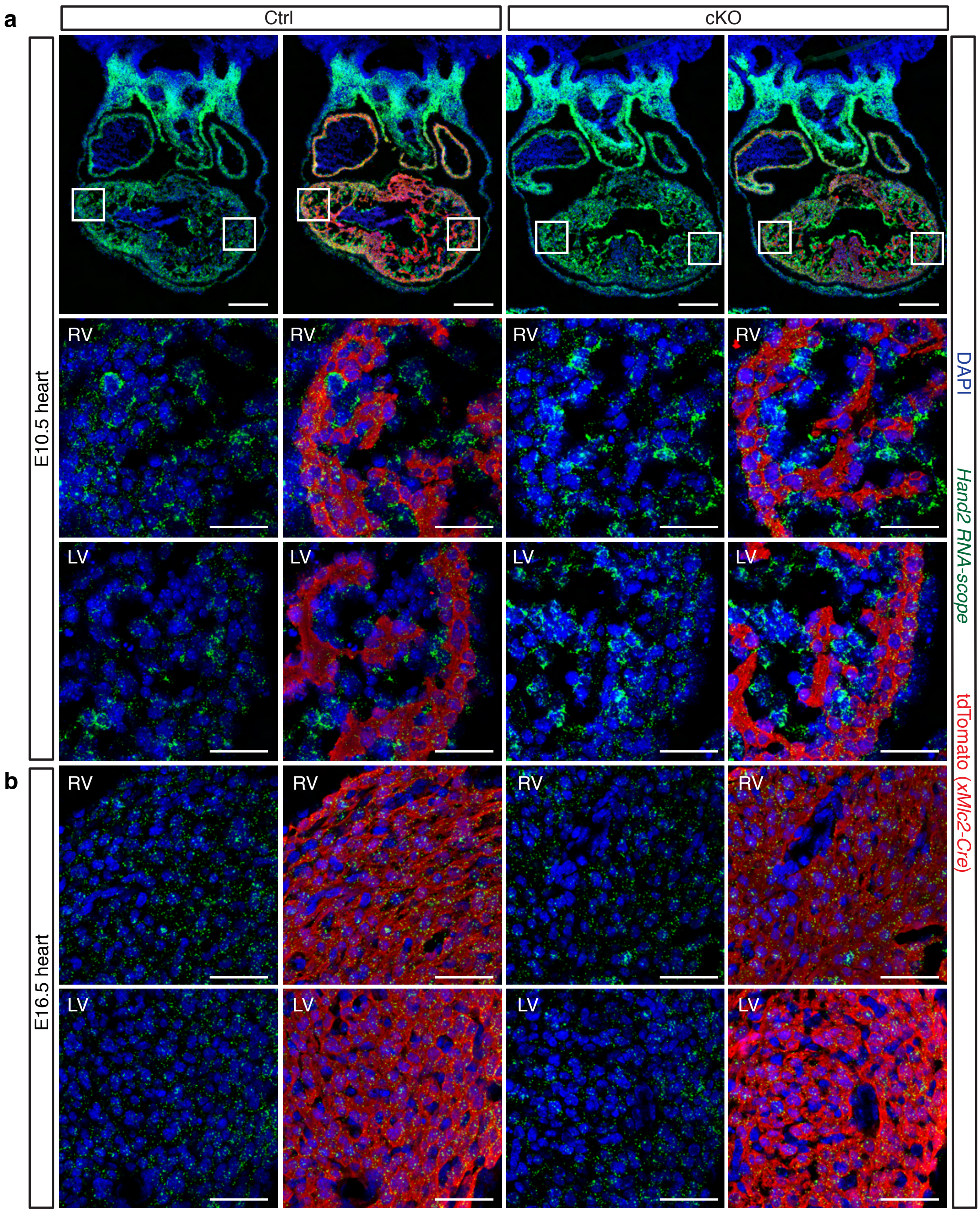

Supplementary Figure 4, related to Figure 3: Normal expression of *Hand2* in Dot1L cKO hearts. a) In control E10.5 hearts, *Hand2* transcripts can be detected in cardiomyocytes from all cardiac chambers and in the endocardium. As shown in the higher magnification of boxed areas, *Hand2* RNA-scope signal was equally abundant in Ctrl and Dot1L cKO hearts (quantification data in Fig. 3f), confirming that expression of this gene is not affected by ablation of DOT1L. **b)** At E16.5 *Hand2* transcript levels were lower than those observed at E10.5. Similar to E10.5, no differences in *Hand2* transcript abundance could be detected between genotype groups. DAPI in blue, RNA-scope signal of *Hand2* probe in green and tdTomato signal driven by *xMlc2*-Cre;Rosa26-tdTomato in red. To allow clear identification of RNA-scope signals, all images are shown both as a 2-color (*Hand2* and DAPI) and 3-color merge. (Scale bar = 250 μm for low magnification panels and 50 μm for high magnification of boxed areas).

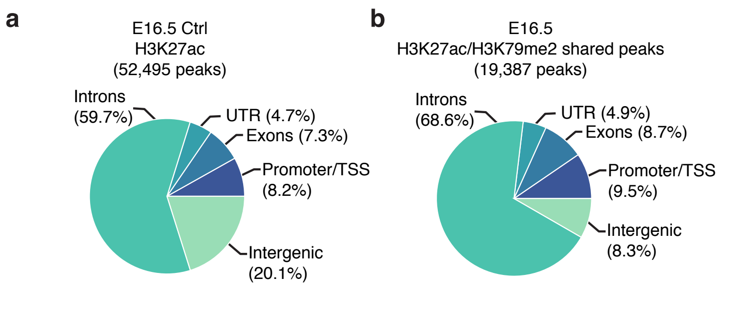

c

**E16.5** (only autosomes)	**All** (N)	**K79-REs** (N)
• **REs**	51,221	19,110
• **REs** with ≥ 1 target gene	47,547	16,963
• **REs** with ≥ 1 target gene that overlap a promoter	10,748	5,715
• **Interactions**	373,610	145,009
• **Interactions** where REs overlaps a promoter	150,001	89,735
• **Interactions** where REs overlaps a promoter of its target gene	13,531	7,639

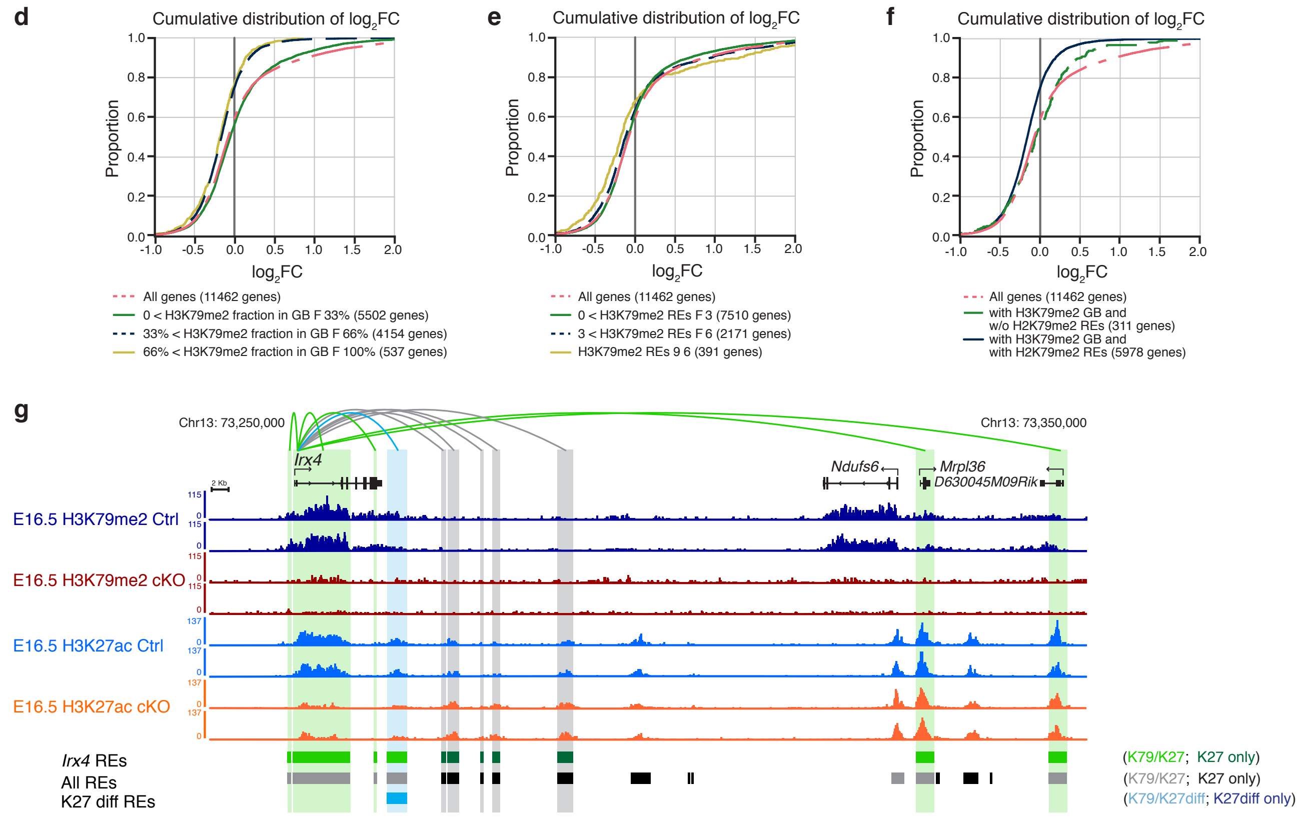

Supplementary Figure 5: a-b) Pie charts indicating the genomic distribution of H3K27ac ChIP-seq peaks (a) and H3K27ac/H3K79me2 shared ChIP-seq peaks (b) in E16.5 Ctrl CMs.
c) Table reporting how often, in E16.5 CMs, REs (top part) or interactions between a REs and its target gene (bottom part) overlap with promoters. All REs are shown on the left and K79-REs are shown on the right (overlap ≥10%). Numbers do not include gonosomes. **d-e-f)** Cumulative log_2FC distribution analyses in different categories of genes in E16.5 CMs: expression changes depending on the the fraction of H3K79me2 coverage in the gene body (GB) (Ctrl coverage ≥1 read) (d); expression changes depending on the number of K79-REs associated with the gene (e); expression changes between genes with gene body H3K79me2 and K79-REs versus genes with gene body H3K79me2 but no K79-REs (f). A Kolmogorov-Smirnov test was applied to assess statistical significance of differences between distribution of gene groups. **g)** Browser tracks displaying H3K79me2 and H3K27ac ChIP-seq profiles of Ctrls (dark and light blue respectively) and Dot1L cKOs (red and orange respectively) E16.5 CMs in the genomic region harboring the *Irx4* locus. Loops display all regulatory interactions between REs and the *Irx4* gene, as identified by the ABC analysis. Gray loops identify interactions with REs without H3K79me2, green loops identify interactions with K79-REs, whereas blue loops represent interactions with K79-REs that additionally have H3K27ac REs differentially enriched between Dot1L Ctrls and cKOs. For reference, all REs are displayed in the middle lane in black or grey, regardless of their regulatory association with the *Irx4* gene. Differential H3K27ac REs are indicated in the bottom lane in light blue when they overlap a K79-RE or dark blue when they do not.

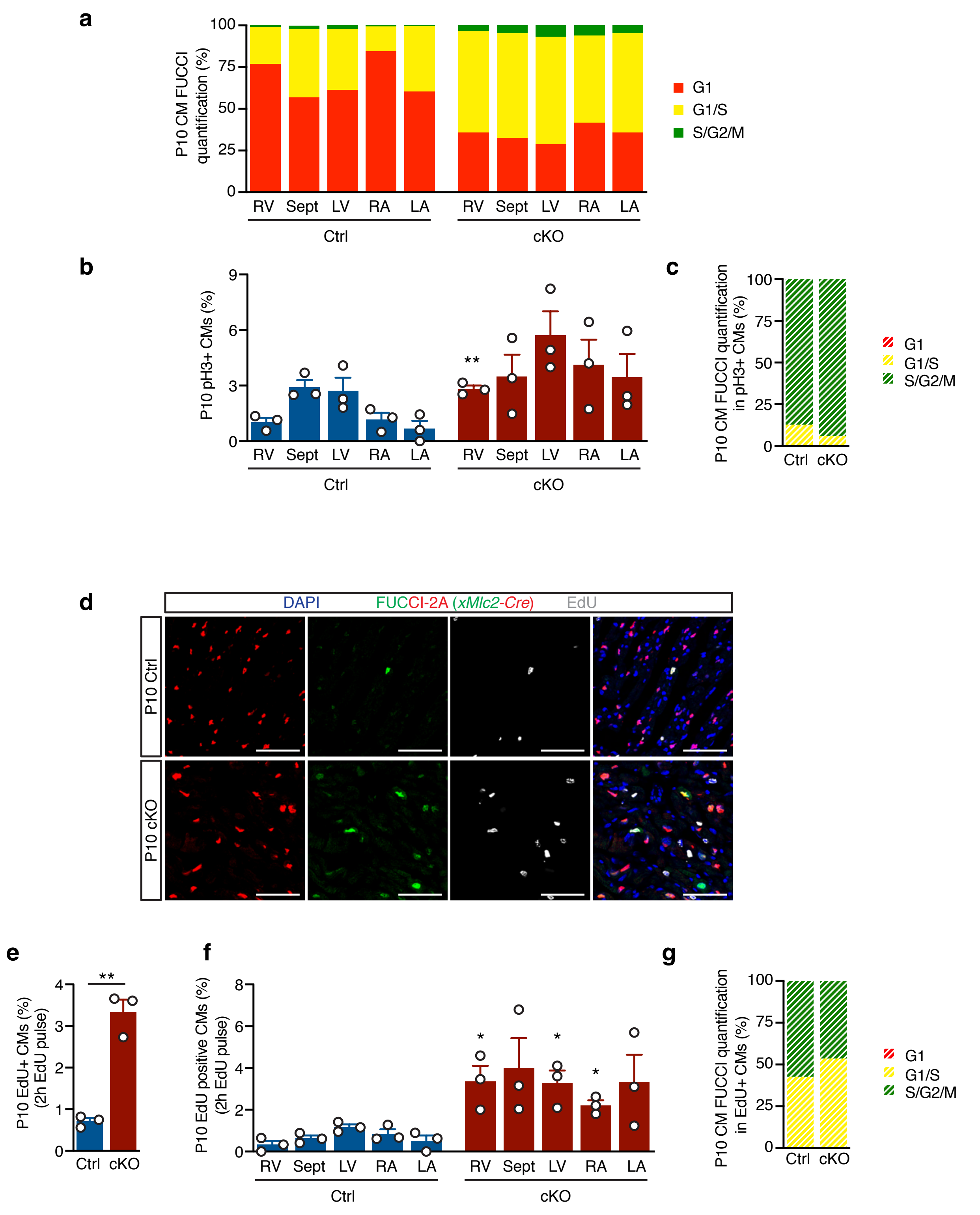

Supplementary Figure 6, related to Figure 6: Dot1L cKO cardiomyocytes fail to undergo neonatal cell cycle withdrawal. a) Quantification of CM cell cycle phase distribution across the distinct cardiac chambers in histological sections from P10 Dot1L Ctrl and cKO hearts on a *Rosa26-Fucci2A* background (Cre-dependent cell cycle indicator). Red only signal represents CMs in G1; red+green (yellow) signal represents CMs in G1/S; green only signal represents CMs in S/G2/M. Right ventricle (RV), septum (Sept), left ventricle (LV), right atrium (RA) and left atrium (LA). Increased ratios of proliferative CMs (G1/S and S/G2/M) were found in all compartments of the Dot1L cKO heart (N=3 biological replicates) **b)** Quantification of mitotic CMs (pH3+) in histological sections of P10 Dot1L Ctrl and cKO hearts (*Rosa26-Fucci2A* background). Compartment-specific

data are presented as percentage of pH3+ CMs over total CMs (N=3 biological replicates; mean ± SEM, unpaired t-test, two-sided RV P=0.0038). **c)** Distribution of pH3+ cells according to the cell cycle stage indicated by the Fucci2A reporter. In both genotype groups the majority of pH3+ CMs corresponded to CMs in S/G2/M (green only) (N=3 biological replicates). **d-e-f)** Immunofluorescence images (d) and corresponding quantification (e,f) showing signals of EdU incorporation (white) on histological sections from P10 Dot1L Ctrl and cKO hearts on a *Rosa26-Fucci2A* indicator background (Scale bars = 50µm). At P10, when analyzing all cardiac chambers together (e) (N=3 biological replicates; unpaired t-test, two-sided P=0.0011), or distinct compartments separately (f), Dot1L cKO hearts displayed significantly higher percentages of EdU+ CMs than their Ctrl counterparts (mean of 1022 CMs counted per heart; N=3 biological replicates; unpaired t-test, two-sided RV P=0.0175, LV P=0.0277, RA P=0.0134). **g)** Distribution of EdU+ cells according to the cell cycle stage indicated by the Fucci2A reporter (N=3 biological replicates). In both genotype groups all EdU+ CMs corresponded to CMs in G1/S (yellow) or S/G2/M (green only). * represents P≤0.05 and ** P≤0.01; Data are presented as mean± SEM. Source data are provided as a Source Data file.

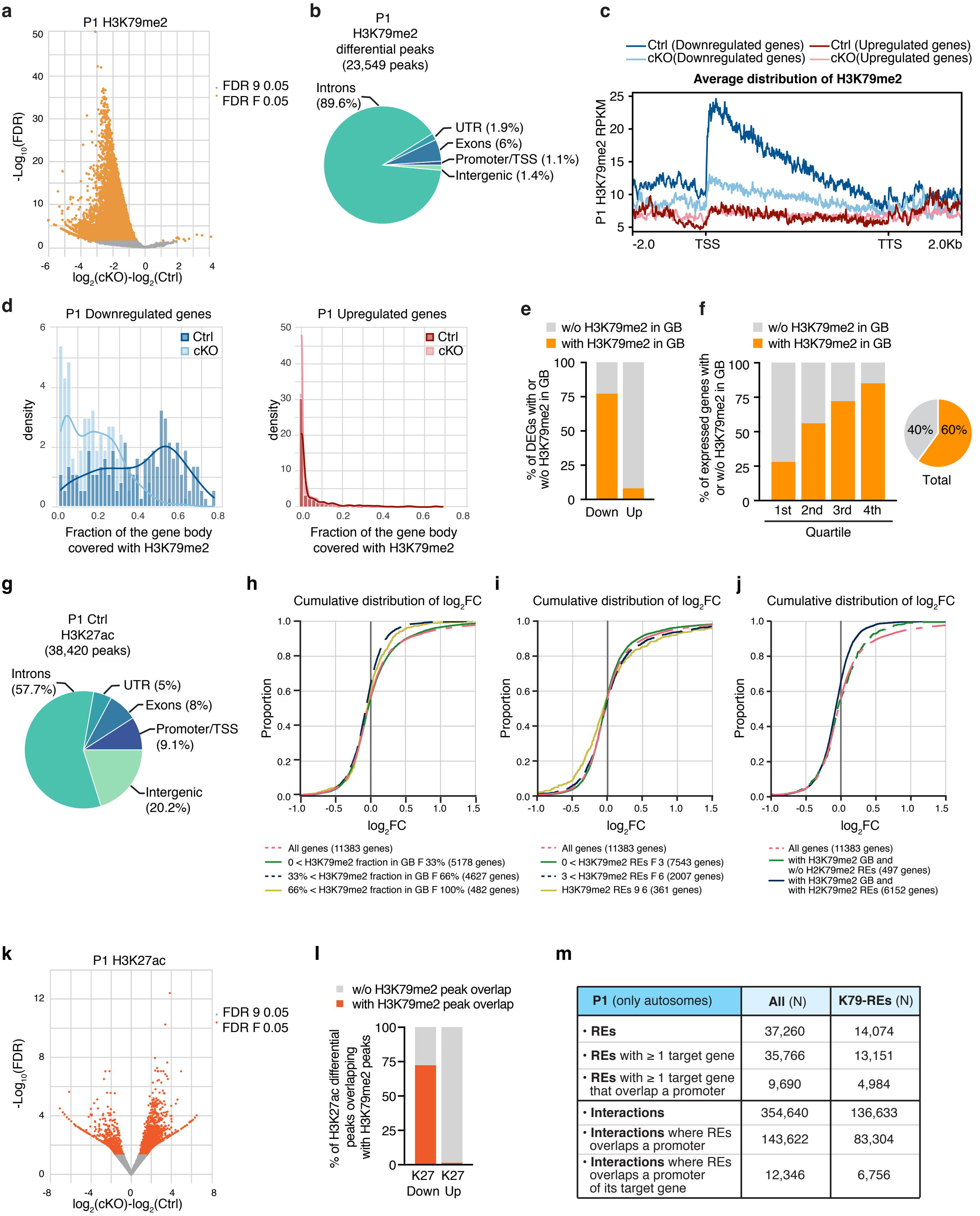

**P1** (only autosomes)	**All** (N)	**K79-REs** (N)
• **REs**	37,260	14,074
• **REs** with ≥ 1 target gene	35,766	13,151
• **REs** with ≥ 1 target gene that overlap a promoter	9,690	4,984
• **Interactions**	354,640	136,633
• **Interactions** where REs overlaps a promoter	143,622	83,304
• **Interactions** where REs overlaps a promoter of its target gene	12,346	6,756

Supplementary Figure 7, related to Figure 7: Mechanisms of gene expression regulation by DOT1L/H3K79me2 in postnatal day 1 cardiomyocytes. a) Volcano plot displaying H3K79me2 ChIP-seq peaks significantly enriched in P1 cKO vs Ctrl CMs. **b)** Pie chart indicating the genomic distribution of differential H3K79me2 ChIP-seq peaks in P1 CMs. **c)** Metagene profiles showing the average distribution of H3K79me2 input-normalized density relative to Transcription Start Site (TSS)

and Transcription Termination Site (TTS) with ±2Kb flanking regions. **d)** Fraction of gene body covered with H3K79me2 in downregulated genes (left graph) and in upregulated genes (right graph) **e)** Graph representing the percentage of down- and up-regulated genes in P1 cKO CMs with (Coverage ≥ 50 reads and Fraction of gene body ≥ 0.2) or without (Coverage < 50 reads or Fraction of gene body < 0.2) gene body H3K79me2 in P1 control CMs. **f)** Percentage of genes with H3K79me2 in the gene body (GB) or without H3K79me2 in the gene body across distinct expression quartiles. **g)** Pie chart indicating the genomic distribution of H3K27ac ChIP-seq peaks in P1 Ctrl CMs. **h-i-j)** Cumulative log2FC distribution analyses in different categories of genes in P1 CMs: expression changes depending on the fraction of H3K79me2 coverage in the gene body (GB) (Ctrl coverage ≥1 read) (d); expression changes depending on the number of K79-REs associated with the gene (e); expression changes between genes with gene body H3K79me2 and K79-REs versus genes with gene body H3K79me2 but no K79-REs (f). A Kolmogorov-Smirnov test was applied to assess statistical significance of differences between distribution of gene groups. **k)** Volcano plot displaying H3K27ac ChIP-seq peaks significantly enriched in P1 cKO vs Ctrl CMs. **l)** Graph representing the percentage of differential H3K27ac ChIP-seq peaks in P1 cKO vs Ctrl CMs overlapping (orange) or not overlapping (grey) with H3K79me2 ChIP-seq peaks. (oddsratio = 0.006; p-value = 0). **m)** Table reporting how often, in P1 CMs, REs (top part) or interactions between a REs and its target gene (bottom part) overlap with promoters. All REs are shown on the left and K79-REs on the right (overlap ≥10%). Numbers do not include gonosomes.

a

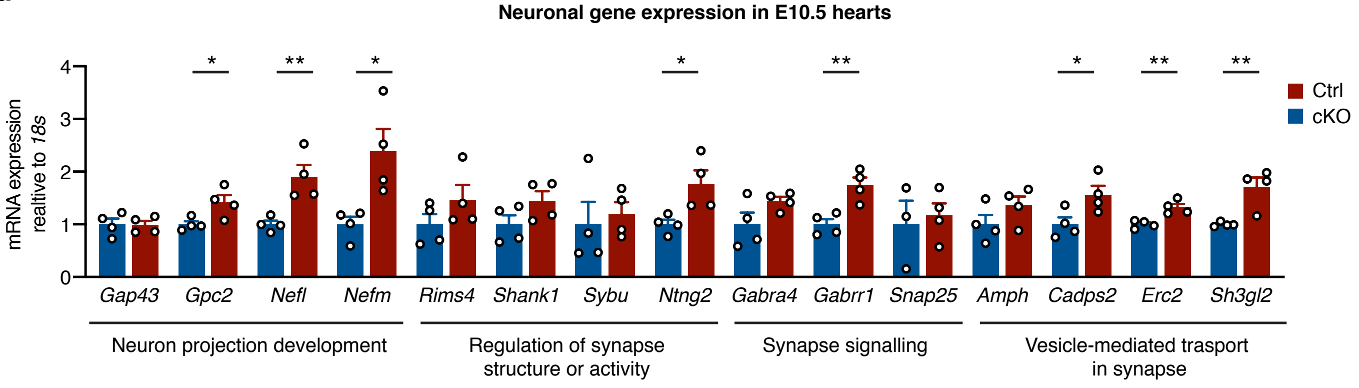

Supplementary Figure 8, related to Figure 7: Upregulation of neuronal genes in Dot1L cKO hearts in early cardiogenesis.

a) qPCR analyses showing that multiple of the neuronal genes upregulated in P1 Dot1L cKO vs Ctrl CMs are already upregulated in early cardiogenesis (E10.5 hearts). (N=4 biological replicates, Data is presented as mean ± SEM; unpaired t-test, two-sided *Gpc2* P=0.033, *Nefl* P=0.009, *Nefm* P=0.022, *Ntng2* P=0.028, *Gabrr1* P=0.006, *Cadps2* P=0.04, *Erc2* P=0.006, *Sh3gl2* P=0.009; * represents P≤0.05, **P≤0.01).
