# Supplementary material for: DOT1L regulates chamber-specific transcriptional networks during cardiogenesis and mediates postnatal cell cycle withdrawal
Source: Nat Commun. 2022 Dec 2;13:7444. doi: 10.1038/s41467-022-35070-2 (PMC9718823; doi:10.1038/s41467-022-35070-2)
Supplement: Supplementary file 3 — Description of Additional Supplementary Files [file 41467_2022_35070_MOESM3_ESM.pdf]

Description of Additional Supplementary Files

File Name: Supplementary Data 1
Description: **Integrated transcriptomics and epigenomics analyses in E16.5 and P1 cardiomyocytes.**
a,b) RNA-seq and H3K79me2 ChIP-seq results comparing the transcriptomes and epigenomes of CMs FACS sorted from E16.5 (a) or P1 (b) Dot1L cKO vs Ctrl hearts. Column headlines and description: *Gene ID, Ensembl ID, UCSC ID* unique identifiers of analyzed genes; *biotype* = category of analyzed genes; *chr, start, end* = coordinates of analyzed genes; *DEG* (Differential Expressed Genes), *logFC* (log Fold Change), *log CPM* (log Count Per Million)*, LR* (Likelihood Ratio) *, PValue, FDR* (False Discovery Rate) refers to the edgeR differential RNA-seq analysis (DOWN = significantly downregulated genes in cKO vs Ctrl with log2FC ≤ 0.5 and FDR ≤ 0.05, UP = significantly upregulated genes in cKO vs Ctrl with log2FC ≥ 0.5; FDR ≤ 0.05, NS = not significantly differentially expressed genes); *Ctrl Coverage, cKO Coverage* = H3K79me2 reads in the gene body in Ctrls and cKOs (mean of replicates); *Ctrl Fraction, cKO Fraction* = fraction of gene body that is covered by H3K79me2 reads in Ctrls and cKOs (corrected for input, mean of replicates).

File Name: Supplementary Data 2
Description: **H3K79me2 ChIP-seq analyses.**
a,b) DiffBind analyses determining differential H3K79me2 ChIP-seq peaks in E16.5 (a) and P1 (b) Dot1L cKO vs Ctrl sorted CMs. Differential bound sites between Ctrl and cKO with FDR≤ 0.05 are shown, sorted for fold enrichment. Column headlines and description: *Chrom, Start, End* = coordinates of analyzed peak; *Width* = Length of site; *Conc* = Mean read concentration over all the samples (the default calculation uses $\log_2$ normalized ChIP read counts with input read counts subtracted); *Conc_Ctrl* = Mean concentration over control group; *Conc_cKO* = Mean concentration over cKO group; *Fold* = difference in mean concentrations between Dot1L Ctrl and Dot1L cKO groups; *p-value* = P-value confidence measure for the identification of individual peaks as differentially bound; *FDR* = False Discovery Rate.

File Name: Supplementary Data 3
Description: **Genomic interactions underlying H3K79me2-dependent gene regulation. a,b)** Element-to-target gene interactions with an adapted Activity-by-contact (ABC) score ≥ 0.02 in E16.5 (a) and P1 (b) CMs. (As indicated in the methods, genes in chromosome X and Y are excluded from the analysis). Column headlines and description: *Peak_Chr, Peak_Start, Peak_End*, refer to the coordinates of each H3K27ac ChIP-seq peak with interactions with target genes; *Interacting geneID* = gene interacting with the peak; *signalValue* = H3K27ac signal value from MACS2; *peakID* = internal ID for identification of H3K27ac peaks; *K79meDiff* = whether or not the H3K27ac peak overlaps with a differential H3K79me2 peak (no=0, yes=1); *K79meFC* = logFoldChange (Dot1L cKO versus Dot1L Ctrl) of the overlapping differential H3K79me2 peak(s) (from DiffBind analysis, Supplementary File 2). If more than one differential H3K79me2 peak overlaps with the H3K27ac peak, the log fold change for each of the H3K79me2 peaks is separated by an "|"; *K27acDiff* and *K27acFC* = same as for K79, fold-change also as cKO versus Ctrl; *adaptedActivity*= peak signal adapted for all gene contacts that the peak forms; *scaledContact* = Contact scaled so that the maximum of all peaks 5Mb around the gene is 100; *ABC-Score* = adapted score of peak-gene interaction; *TSS-dist* = distance between the peak and

the transcriptional start site (TSS) of its target gene; *ovPromoter* = if the H3K27ac peak overlaps with a promoter of a known gene, the ID of such gene(s) is listed in this column; *Location* = genomic location of the peak. In this classification "1.0_intergenic" means the peak does not overlap with any annotated gene. "1.0_genebody" means the peak is 100% within a gene. In this case, additional information is provided as to peak distribution across specific gene domains (promoter/exon/UTR). The gene regions are not exclusive.

File Name: Supplementary Data 4
Description: **Number of REs associated to gene expression. a)** Analysis in E16.5 CMs **b)** Analysis in P1 CMs. (As indicated in the methods, genes in chromosome X and Y are excluded from the analysis). Column headlines and description: *geneID, GeneName, chr,* and *TSS* (Transcription Start Site) refer to each gene expressed in FACS-sorted CMs (E16.5, or P1, depending on the tab) and having at least one element-to-target gene interaction identified by the ABC analysis; *DEG, , logFC, FDR* refers to the RNA-seq analysis (DOWN = significantly downregulated in cKO vs Ctrl with $\log_2$FC ≤ - 0.5 and FDR ≤ 0.05, UP = significantly upregulated in cKO vs Ctrl with $\log_2$FC ≥ 0.5; FDR ≤ 0.05, NS = not significantly differentially expressed); *Ctrl Coverage, cKO Coverage* = H3K79me2 reads in the gene body in Ctrls and cKOs (mean of replicates); *Ctrl Fraction, cKO Fraction* = fraction of gene body that is covered by H3K79me2 reads in Ctrls and cKOs corrected for input (mean of replicates); H3K79me2_GB = presence or absence of H3K79me2 in gene body (Ctrl coverage ≥ 50 and Ctrl fraction ≥ 0.2) (Y = yes, N = no); *#All-K27-REs* = number of all associated regulatory elements; *#All-K27/K79-REs* = number of associated regulatory elements that overlap a differential peak for H3K79me2; *#Intragenic-K27-REs* = number of regulatory elements that are located intragenic; *#Intragenic-K27/K79-REs* = number of intragenic regulatory elements that overlap a differential peak for H3K79me2; *#Intergenic-K27-REs* = number of regulatory elements that are located intergenic; *#Intergenic-K27/K79-REs* = number of intergenic regulatory elements that overlap a differential peak for H3K79me2); *#K27acDiff-REs, #K27Down-REs, #K27Up-REs* = number of associated regulatory elements that are differential for H3K27ac (cKO vs Ctrl), downregulated and upregulated respectively.

File Name: Supplementary Data 5
Description: **Metascape pathway analyses.** Analysis in E16.5 (a,b) and P1 (c,d) CMs of metascape categories enriched for genes downregulated with H3K79me2 in GB and REs (a, c) and upregulated with H3K79me2 in REs (b, d) in Dot1L cKO vs Ctrl CMs.

File Name: Supplementary Data 6
Description: **H3K27ac ChIP-seq analyses.**
a,b) DiffBind analyses determining differential H3K27ac ChIP-seq peaks in E16.5 (a) and P1 (b) Dot1L cKO vs Ctrl sorted CMs. Differential bound sites between Ctrl and cKO with FDR≤ 0.05 are shown, sorted for fold enrichment. Column headlines and description: *Chrom, Start, End* = coordinates of analyzed peak; *Width* = Length of site; *Conc* = Mean read concentration over all the samples (the default calculation uses log2 normalized ChIP read counts with input read counts subtracted); *Conc_Ctrl* = Mean concentration over control group; *Conc_cKO* = Mean concentration over cKO group; *Fold* = difference in mean concentrations between Dot1L Ctrl and Dot1L cKO groups; *p-value* = P-value confidence measure for the identification of individual peaks as differentially bound; *FDR* = False Discovery Rate.
