# Supplementary material for: DOT1L regulates chamber-specific transcriptional networks during cardiogenesis and mediates postnatal cell cycle withdrawal
Source: Nat Commun. 2022 Dec 2;13:7444. doi: 10.1038/s41467-022-35070-2 (PMC9718823; doi:10.1038/s41467-022-35070-2)
Supplement: Supplementary file 10 — Reporting Summary [file 41467_2022_35070_MOESM10_ESM.pdf]

nature portfolio

Corresponding author(s): Sylvia M. Evans
Paola Cattaneo

Last updated by author(s): Nov 10, 2022

Reporting Summary

Nature Portfolio wishes to improve the reproducibility of the work that we publish. This form provides structure for consistency and transparency in reporting. For further information on Nature Portfolio policies, see our Editorial Policies and the Editorial Policy Checklist.

Statistics

For all statistical analyses, confirm that the following items are present in the figure legend, table legend, main text, or Methods section.

n/a	Confirmed	
☐	☒	The exact sample size (*n*) for each experimental group/condition, given as a discrete number and unit of measurement
☐	☒	A statement on whether measurements were taken from distinct samples or whether the same sample was measured repeatedly
☐	☒	The statistical test(s) used AND whether they are one- or two-sided<br>*Only common tests should be described solely by name; describe more complex techniques in the Methods section.*
☒	☐	A description of all covariates tested
☒	☐	A description of any assumptions or corrections, such as tests of normality and adjustment for multiple comparisons
☐	☒	A full description of the statistical parameters including central tendency (e.g. means) or other basic estimates (e.g. regression coefficient) AND variation (e.g. standard deviation) or associated estimates of uncertainty (e.g. confidence intervals)
☐	☒	For null hypothesis testing, the test statistic (e.g. *F*, *t*, *r*) with confidence intervals, effect sizes, degrees of freedom and *P* value noted<br>*Give P values as exact values whenever suitable.*
☒	☐	For Bayesian analysis, information on the choice of priors and Markov chain Monte Carlo settings
☒	☐	For hierarchical and complex designs, identification of the appropriate level for tests and full reporting of outcomes
☒	☐	Estimates of effect sizes (e.g. Cohen's *d*, Pearson's *r*), indicating how they were calculated

Our web collection on statistics for biologists contains articles on many of the points above.

Software and code

Policy information about availability of computer code

Data collection	No software was used for data collection
Data analysis	DIVA (8.0.1) (BD Pharmingen), FlowJo (10.8.1) software (BD Pharmingen), STAR (v.2.5.1b), edgeR, METASCAPE, Bowtie2, deeptools, MACS2 (version macs2 2.2.7.1), DiffBind (version 2.10.0 and version 3.4.11 bioconductor-diffbind), bedtools (v2.25.0), Juicebox dump command (version 1.22.01), TRAP, Integrative Genomics Viewer (IGV) (v2.9.2), Prism 8, VevoLab 3.2.5 software, ImageJ, Volocity

For manuscripts utilizing custom algorithms or software that are central to the research but not yet described in published literature, software must be made available to editors and reviewers. We strongly encourage code deposition in a community repository (e.g. GitHub). See the Nature Portfolio guidelines for submitting code & software for further information.

Data

Policy information about availability of data

All manuscripts must include a data availability statement. This statement should provide the following information, where applicable:

- Accession codes, unique identifiers, or web links for publicly available datasets
- A description of any restrictions on data availability
- For clinical datasets or third party data, please ensure that the statement adheres to our policy

All RNA-seq and ChIP-seq data that support the findings of this study have been deposited in the Gene Expression Omnibus (GEO) under accession code GSE184192 (https://www.ncbi.nlm.nih.gov/geo/query/acc.cgi?acc=GSE184192). Previously published ChIP-seq and Hi-C data that were re-analyzed here are available under accession code GSM2544836 (https://www.ncbi.nlm.nih.gov/geo/query/acc.cgi?acc= GSM2544836) from GEO. Source data are provided with this paper.

Field-specific reporting

Please select the one below that is the best fit for your research. If you are not sure, read the appropriate sections before making your selection.

☒ Life sciences ☐ Behavioural & social sciences ☐ Ecological, evolutionary & environmental sciences

For a reference copy of the document with all sections, see nature.com/documents/nr-reporting-summary-flat.pdf

Life sciences study design

All studies must disclose on these points even when the disclosure is negative.

Sample size	Sample size was determined on calculations based on our previous published data (https://doi.org/10.1016/j.devcel.2015.04.021), showing that a sample size of n = 3 to 5 mice per group allows detection of the (large) effect sizes of interest with a probability greater than 0.9. Sample size for next generation sequencing data were determined following ENCODE consortium guidelines. For RNA-seq experiments cardiomyocytes from three hearts per genotype were used from biological replicates. For ChIP-seq experiments, two replicates, each including cardiomyocytes pooled from at least two individual mouse heart were used. For echocardiografic analyses 11 to 13 biological replicates were used per genotype. For RNA-scope and Immunohistochemistry 3 biological replicates were used for each genotype. Quantification of EdU, pH3 and Fucci signal in histological sections of P10 hearts, cells was done in at least three biological replicates per genotype group. For each biological replicate, cells were manually quantified from eight randomly selected field from left ventricle, right ventricle, septum and four randomly selected field from left atria and right atria.
Data exclusions	No data were excluded.
Replication	All attempts of replication were successful. Each experiment was repeated independently with similar results at least 2-3 times.
Randomization	Experimental groups were determined by genotype (i.e. Dot1L control embryos/pups were compared to Dot1L conditional KO embryos/pups).
Blinding	Investigators were not blinded to allocation of embryos/mice for experimental groups because this choice was driven by genotype. Experiments that were consistently blinded included immunohistochemistry, RNA-scope, FACS and qPCR and echocardiographic analyses.

Reporting for specific materials, systems and methods

We require information from authors about some types of materials, experimental systems and methods used in many studies. Here, indicate whether each material, system or method listed is relevant to your study. If you are not sure if a list item applies to your research, read the appropriate section before selecting a response.

Materials & experimental systems

n/a	Involved in the study	
☐	☒	Antibodies
☒	☐	Eukaryotic cell lines
☒	☐	Palaeontology and archaeology
☐	☒	Animals and other organisms
☒	☐	Human research participants
☒	☐	Clinical data
☒	☐	Dual use research of concern

Methods

n/a	Involved in the study	
☐	☒	ChIP-seq
☐	☒	Flow cytometry
☒	☐	MRI-based neuroimaging

Antibodies

Antibodies used	anti-aSarcomeric Actinin (Abcam #ab68167), anti-Vimentin (Abcam#ab45939), anti-TNNT (Thermo Fisher #MA5-12960), anti-PDGFR-a (R&D Systems #AF1062), anti-tdTomato (Sicgen #ab8181-200), anti-GFP (Abcam #ab13970), anti-phosphoH3 (Millipore #06-570), H3K79me2 (abcam #ab3594), anti-H3K27ac (Active Motif #39133) anti-H3 (abcam #ab1791), anti-Myomesin (mMaC, Developmental Studies Hybridoma Bank #B4), anti-Collagen 1 (abcam #ab34710), anti-CD31 (clone: MEC13.3; BioLegend #102510), anti-CD45 (clone: 30-F11, BioLegend #103112), anti-CD140a (clone: APA5, eBioscience #17-1401-81), anti-TER119 (clone: TER-119, BioLegend #116212), Rabbit HRP-conjugated secondary antibody (Cell Signaling Technology #7074), donkey anti-rabbit Alexa fluor 647 (Thermofisher #A31573), donkey anti-goat Alexa fluor 488 (Thermofisher #A11055), donkey anti-goat Alexa fluor 555 (Thermofisher #A21432), donkey anti-chicken Alexa fluor 488 (Jackson Immuno Research #703-545-155), donkey anti-rabbit Alexa fluor 555 (Thermofisher #A31572), donkey anti-mouse Alexa fluor 488 (Thermofisher #A21202), donkey anti-goat Alexa fluor 647 (Thermofisher #A21447)
Validation	All antibodies used in our study are commercially purchased from credible sources and have been validated by the manufacturer. All antibodies used have been cited several times by other investigator and tested on our positive control samples.<br>anti-aSarcomeric Actinin validated on mouse heart and skeletal muscle (https://www.abcam.com/sarcomeric-alpha-actinin-antibody-ep2529y-ab68167.html).<br>anti-Vimentin validated in mouse kidney (https://www.abcam.com/vimentin-antibody-cytoskeleton-marker-ab45939.html).

anti-TNNT validated in mouse heart (https://www.thermofisher.com/antibody/product/Cardiac-Troponin-T-Antibody-clone-13-11-Monoclonal/MA5-12960).
anti-tdTomato validated in brain (https://www.sicgen.pt/product/tdtomato-polyclonal-antibody_1_135) and in positive and negative controls of our tissues samples.
anti-GFP validated in spinal cord (https://www.abcam.com/gfp-antibody-ab13970.html) and in positive and negative controls of our tissue samples.
anti-PhospohoH3 validated in Hela Cells (https://www.merckmillipore.com/DE/en/product/Anti-phospho-Histone-H3-Ser10-Antibody-Mitosis-Marker,MM_NF-06-570?ReferrerURL=https%3A%2F%2Fwww.google.com%2F).
anti-H3K79me2 validated in Hela Cells (https://www.abcam.com/histone-h3-di-methyl-k79-antibody-chip-grade-ab3594.html).
anti-H3K27ac validated in HAP1 myeloid leukemia cell (https://www.activemotif.com/catalog/details/39133/histone-h3-acetyl-lys27-antibody-pab).
anti-H3 validated in Hela Cells (https://www.abcam.com/Histone-H3-antibody-Nuclear-Marker-and-ChIP-Grade-ab1791.html?gclsrc=aw.ds%7Caw.ds&gclid=Cj0KCQiA37KbBhDgARIsAIzce15_V8A29bQ03GMw8ito1R4H77zUexOqLd3A1r596DWB0dOWHcE2NGUaAgcUEALw_wcB).
anti-Myomesin validated in heart and skeletal muscle (https://dshb.biology.uiowa.edu/mMaC-myomesin-B4).
anti-Collagen 1 validated in heart (https://www.abcam.com/collagen-i-antibody-ab34710.html).
anti-CD31 validated in mouse splenocytes (https://www.biolegend.com/nl-be/products/pe-anti-mouse-cd31-antibody-379).
anti-CD45 validated in mouse splenocytes (https://www.biolegend.com/nl-be/products/apc-anti-mouse-cd45-antibody-97).
anti-CD140a validated in NIH-3T3 (https://www.thermofisher.com/antibody/product/CD140a-PDGFRA-Antibody-clone-APA5-Monoclonal/17-1401-81).
anti-TER119 validated in bone marrow (https://www.biolegend.com/nl-be/products/apc-anti-mouse-ter-119-erythroid-cells-antibody-1863).

Animals and other organisms

Policy information about studies involving animals; ARRIVE guidelines recommended for reporting animal research

Laboratory animals

Mice included in this study were kept on an outbread background (Black-Swiss, Charles River laboratories). Mice were maintained in plastic cages with filtered air intake ports on a 12 hr light cycle and monitored daily with no health problems reported. All animals were housed in groups of maximum five per cage with ad libitum access to food and water. Adult (2-12 months old) males and females were used for breeding. Experimental mice (males and females) were analyzed from embryonic day E10.5 to P10. Dot1lflox mice were obtained from the KOMP Repository (CSD29070). xMlc2-Cre mice were gently provided by Timothy Mohun. The Rosa26-tdTomato (Ai9) (tdTom) indicator allele was purchased from JAX (Stock No: 007905). The Rosa26-Fucci2A cell cycle reporter allele was gently provided by Ian James Jackson. Meox2-Cre mice were obtained from JAX laboratories (Stock No: 026858).

Wild animals

The study did not involve wild animals.

Field-collected samples

The study did not involve field-collected samples.

Ethics oversight

All experiments involving animals were approved by the Institutional Animal Care and Use Committee at University of California, San Diego and the German local ethic committee (Regierungspräsidium Darmstadt, Hessen). All procedures were performed in accordance with the National Institute of Health Guide for the Dare and Use of Laboratory Animals.

Note that full information on the approval of the study protocol must also be provided in the manuscript.

ChIP-seq

Data deposition

☒ Confirm that both raw and final processed data have been deposited in a public database such as GEO.

☒ Confirm that you have deposited or provided access to graph files (e.g. BED files) for the called peaks.

Data access links
May remain private before publication.

The next generation sequencing data that support the findings of this study have been deposited in GEO GSE184192 with full access available to reviewers using token wzyheegsvjgxvit. Public access will be enabled upon publication. All data will be available upon request.

Files in database submission

GSM5578010 CM_E16_Ctrl_rep1_H3K79me2 (ChIP-seq fastq and bigWig)
GSM5578011 CM_E16_Ctrl_rep1_input (ChIP-seq fastq)
GSM5578012 CM_E16_Ctrl_rep2_H3K79me2 (ChIP-seq fastq and bigWig)
GSM5578013 CM_E16_Ctrl_rep2_input (ChIP-seq fastq)
GSM5578014 CM_E16_cKO_rep1_H3K79me2 (ChIP-seq fastq and bigWig)
GSM5578015 CM_E16_cKO_rep1_input (ChIP-seq fastq)
GSM5578016 CM_E16_cKO_rep2_H3K79me2 (ChIP-seq fastq and bigWig)
GSM5578017 CM_E16_cKO_rep2_input (ChIP-seq fastq)
GSM5578018 CM_P1_Ctrl_rep1_H3K79me2 (ChIP-seq fastq and bigWig)
GSM5578019 CM_P1_Ctrl_rep1_input (ChIP-seq fastq)
GSM5578020 CM_P1_Ctrl_rep2_H3K79me2 (ChIP-seq fastq and bigWig)
GSM5578021 CM_P1_Ctrl_rep2_input (ChIP-seq fastq)
GSM5578022 CM_P1_cKO_rep1_H3K79me2 (ChIP-seq fastq and bigWig)
GSM5578023 CM_P1_cKO_rep1_input (ChIP-seq fastq)
GSM5578024 CM_P1_cKO_rep2_H3K79me2 (ChIP-seq fastq and bigWig)
GSM5578025 CM_P1_cKO_rep2_input (ChIP-seq fastq)

GSM6471479 CM_E16_Dot1L_Ctrl_rep1_H3K27ac_ChIP (ChIP-seq fastq and bigWig)
GSM6471480 CM_E16_Dot1L_Ctrl_rep1_H3K27ac_input (ChIP-seq fastq and bigWig)
GSM6471482 CM_E16_Dot1L_Ctrl_rep2_H3K27ac_ChIP (ChIP-seq fastq and bigWig)
GSM6471483 CM_E16_Dot1L_Ctrl_rep2_H3K27ac_input (ChIP-seq fastq and bigWig)
GSM6471485 CM_E16_Dot1L_cKO_rep1_H3K27ac_ChIP (ChIP-seq fastq and bigWig)
GSM6471486 CM_E16_Dot1L_cKO_rep1_H3K27ac_input (ChIP-seq fastq and bigWig)
GSM6471488 CM_E16_Dot1L_cKO_rep2_H3K27ac_ChIP (ChIP-seq fastq and bigWig)
GSM6471489 CM_E16_Dot1L_cKO_rep2_H3K27ac_input (ChIP-seq fastq and bigWig)
GSM6471491 CM_P1_Dot1L_Ctrl_rep1_H3K27ac_ChIP (ChIP-seq fastq and bigWig)
GSM6471492 CM_P1_Dot1L_Ctrl_rep2_H3K27ac_ChIP (ChIP-seq fastq and bigWig)
GSM6471494 CM_P1_Dot1L_Ctrl_rep1-2_H3K27ac_input (ChIP-seq fastq and bigWig)
GSM6471495 CM_P1_Dot1L_cKO_rep1_H3K27ac_ChIP (ChIP-seq fastq and bigWig)
GSM6471497 CM_P1_Dot1L_cKO_rep2_H3K27ac_ChIP (ChIP-seq fastq and bigWig)
GSM6471498 CM_P1_Dot1L_cKO_rep1-2_H3K27ac_input (ChIP-seq fastq and bigWig)

Genome browser session (e.g. UCSC)
N/A

Methodology

Replicates
ChIP-seq experiments were conducted with n=2 biological replicates with satisfactory agreement by Principal Component Analysis.

Sequencing depth
For H3K79me2 ChIP-seq libraries were sequenced single-end for 75 cycles (SE75) to a depth of 20-25 million reads on an Illumina NextSeq 500 instrument. For H3K27ac ChIP-seq libraries were sequenced single-end for 76 cycles (SE76) to a depth of 15-22 million reads on an Illumina NextSeq 500 instrument.

Antibodies
The anti-H3K79me2 antibody (Abcam #ab3594) and anti-H3K27ac anitibody (Active Motif #39133, lot 31521015) were used for ChIP-seq experiments

Peak calling parameters
Bowtie243 was applied to align the fastq files to the mouse reference genome. First, the required index structure was built using: bowtie2-build -f --seed 123 --threads 20 Mus_musculus.GRCm38.dna.primary_assembly.fa mouse_GRCM38_mm10. Since the reads where not paired, we ran for each fastq file: bowtie2 -x mouse_GRCM38_mm10 -U <fastq-file> -S <output-file-name>.sam -q -t -p 30. Conversion of the resulting files (sam) to bam format was done using samtools (version samtools 1.10)44. Next peak-calling was performed with MACS2 (version macs2 2.2.7.1): macs2 callpeak -t <treatment>.bam -c <input>.bam -n <prefix-name> --outdir <output-dir> -f BAM -g 1.87e9 -B, where <treatment> is either the aligned reads of the Dot1L Ctrl or Dot1L cKO and <input> the corresponding input signal.

Data quality
Data quality was checked using FastQC

Software
Bowtie2, deeptools, MACS2 (version macs2 2.2.7.1), DiffBind (version 2.10.0 and version 3.4.11 bioconductor-diffbind), bedtools (v2.25.0).

Flow Cytometry

Plots

Confirm that:

☒ The axis labels state the marker and fluorochrome used (e.g. CD4-FITC).

☒ The axis scales are clearly visible. Include numbers along axes only for bottom left plot of group (a 'group' is an analysis of identical markers).

☒ All plots are contour plots with outliers or pseudocolor plots.

☒ A numerical value for number of cells or percentage (with statistics) is provided.

Methodology

Sample preparation
Single cell suspensions from the heart were obtained by performing enzymatic digestions with Trypsin and Collagenase type II. After digestion, cells were collected in cold medium containing fetal calf serum to stop the enzymatic reaction and resuspended in FACS buffer. EdU detection was done in cell suspensions using the Click-iT® EdU Alexa 647 kit (Thermo Fisher Scientific; C10340), according to the manufacturer's instructions. TdTomato signal was used to discriminate cardiomyocytes from other lineages of cardiac cells. Cells were analyzed using FACS Canto II flow cytometer (BD Bioscience), DIVA and FlowJo software (BD Pharmingen).

Instrument
FACS Canto II flow cytometer (BD Bioscience).

Software
DIVA and FlowJo software (BD Pharmingen) were used for data acquisition and analysis.

Cell population abundance
Following live cell gating and single cell gating, from all the cardiac cells ~50% were tdTomato+ (cardiomyocytes). From these ~18-45% were EdU+.

Gating strategy
Following for FSC/SSC for live cell gating and doublet exclusions, cells were first gated based on the endogenous tdTomato

Gating strategy

signal to identify cardiomyocytes and then on the EdU-647 signal by gating positive cell populations.

☒ Tick this box to confirm that a figure exemplifying the gating strategy is provided in the Supplementary Information.
